# Phylogroup stability contrasts with high within sequence type complex dynamics of *Escherichia coli* bloodstream infection isolates over a 12-year period

**DOI:** 10.1186/s13073-021-00892-0

**Published:** 2021-05-05

**Authors:** Guilhem Royer, Mélanie Mercier Darty, Olivier Clermont, Bénédicte Condamine, Cédric Laouenan, Jean-Winoc Decousser, David Vallenet, Agnès Lefort, Victoire de Lastours, Erick Denamur, COLIBAFI and SEPTICOLI groups, Michel Wolff, Loubna Alavoine, Xavier Duval, David Skurnik, Paul-Louis Woerther, Antoine Andremont, Etienne Carbonnelle, Olivier Lortholary, Xavier Nassif, Sophie Abgrall, Françoise Jaureguy, Bertrand Picard, Véronique Houdouin, Yannick Aujard, Stéphane Bonacorsi, Agnès Meybeck, Guilène Barnaud, Catherine Branger, Agnès Lefort, Bruno Fantin, Claire Bellier, Frédéric Bert, Marie-Hélène Nicolas-Chanoine, Bernard Page, Julie Cremniter, Jean-Louis Gaillard, Françoise Leturdu, Jean-Pierre Sollet, Gaëtan Plantefève, Xavière Panhard, France Mentré, Estelle Marcault, Florence Tubach, Virginie Zarrouk, Frederic Bert, Marion Duprilot, Véronique Leflon-Guibout, Naouale Maataoui, Laurence Armand, Liem Luong Nguyen, Rocco Collarino, Anne-Lise Munier, Hervé Jacquier, Emmanuel Lecorché, Laetitia Coutte, Camille Gomart, Ousser Ahmed Fateh, Luce Landraud, Jonathan Messika, Elisabeth Aslangul, Magdalena Gerin, Alexandre Bleibtreu, Mathilde Lescat, Violaine Walewski, Frederic Mechaï, Marion Dollat, Anne-Claire Maherault, Michel Wolff, Mélanie Mercier-Darty, Bernadette Basse

**Affiliations:** 1 Université de Paris, IAME, UMR 1137, INSERM, F-75018 Paris, France; 2 LABGeM, Génomique Métabolique, Genoscope, Institut François Jacob, CEA, CNRS, Université Paris-Saclay, Evry, France; 3 Département de Prévention, Diagnostic et Traitement des Infections, Hôpital Henri Mondor, F-94000 Créteil, France; 4 Département d’épidémiologie, biostatistiques et recherche clinique, Hôpital Bichat, AP-HP, F-75018 Paris, France; 5 Service de Médecine Interne, Hôpital Beaujon, AP-HP, F-92100 Clichy, France; 6 Laboratoire de Génétique Moléculaire, Hôpital Bichat, AP-HP, F-75018 Paris, France

**Keywords:** Escherichia coli, Bloodstream infection, Antibiotic resistance, Extra-intestinal infection, Pandemic clones, Clonal replacement

## Abstract

**Background:**

*Escherichia coli* is the leading cause of bloodstream infections, associated with a significant mortality. Recent genomic analyses revealed that few clonal lineages are involved in bloodstream infections and captured the emergence of some of them. However, data on within sequence type (ST) population genetic structure evolution are rare.

**Methods:**

We compared whole genome sequences of 912 *E*. *coli* isolates responsible for bloodstream infections from two multicenter clinical trials that were conducted in the Paris area, France, 12 years apart, in teaching hospitals belonging to the same institution (“Assistance Publique-Hôpitaux de Paris”). We analyzed the strains at different levels of granularity, i.e., the phylogroup, the ST complex (STc), and the within STc clone taking into consideration the evolutionary history, the resistance, and virulence gene content as well as the antigenic diversity of the strains.

**Results:**

We found a mix of stability and changes overtime, depending on the level of comparison. Overall, we observed an increase in antibiotic resistance associated to a restricted number of genetic determinants and in strain plasmidic content, whereas phylogroup distribution and virulence gene content remained constant. Focusing on STcs highlighted the pauci-clonality of the populations, with only 11 STcs responsible for more than 73% of the cases, dominated by five STcs (STc73, STc131, STc95, STc69, STc10). However, some STcs underwent dramatic variations, such as the global pandemic STc131, which replaced the previously predominant STc95. Moreover, within STc131, 95 and 69 genomic diversity analysis revealed a highly dynamic pattern, with reshuffling of the population linked to clonal replacement sometimes coupled with independent acquisitions of virulence factors such as the *pap* gene cluster bearing a *papGII* allele located on various pathogenicity islands. Additionally, STc10 exhibited huge antigenic diversity evidenced by numerous O:H serotype/*fimH* allele combinations, whichever the year of isolation.

**Conclusions:**

Altogether, these data suggest that the bloodstream niche is occupied by a wide but specific phylogenetic diversity and that highly specialized extra-intestinal clones undergo frequent turnover at the within ST level. Additional worldwide epidemiological studies overtime are needed in different geographical and ecological contexts to assess how generalizable these data are.

**Supplementary Information:**

The online version contains supplementary material available at 10.1186/s13073-021-00892-0.

## Background

*Escherichia coli* bloodstream infections represent a considerable and increasing burden in human medicine [1] due to the increase both in incidence of the disease and antibiotic resistance of the strains [2]. The in-hospital mortality of these bloodstream infections varies between 10 and 20% in highly developed countries [3–6], and the determinants associated with death are still debated. A major role has been attributed to host conditions (mainly comorbidities including immunosuppression) and to the portal of entry, the urinary one being protective [3–9]. However, several studies have also pointed the role of bacterial characteristics such as clonal group belonging and presence of specific virulence genes [3, 5, 6, 8, 9], whereas the impact of antibiotic resistance is unclear [4–6, 10].

The reservoir of *E*. *coli* strains involved in extra-intestinal infections including bloodstream infections is the gut, where they behave as commensals [11]. The extra-intestinal intrinsic virulence of *E*. *coli* strains has been evaluated thoroughly using a mouse model of sepsis [12–14] and is linked to the phylogenetic/clonal background (mainly phylogenetic group B2 associated to increased virulence) and the presence of virulence genes encoding for iron capture systems, protectins, invasins, adhesins, and toxins. Of note, no evidence has been found that this intrinsic virulence is associated with patients’ death [15]. It has been suggested that these virulence factors (VFs) may rather have been selected for their advantages in the commensal niche where virulence may be considered as a by-product of commensalism [16].

*E*. *coli* epidemiology has changed during the last 20 years with the emergence of the sequence type (ST) 131 clone/clonal complex (CC) belonging to the B2 phylogroup that has disseminated worldwide and is often associated with multidrug resistance [17]. Because of the geographic component of *E*. *coli* epidemiology, at least for commensal strains [11], the understanding of the epidemiologic evolution of *E*. *coli* strains requires studies performed at a local level which are rare. Our group has compared commensal *E*. *coli* strains in the Paris area between 1980 and 2010 and showed a substantial increase in B2 phylogroup strains and VF content as well as antibiotic resistance [18]. Two studies have described the population structure of *E*. *coli* causing bloodstream infections between 2001 and 2012 in the UK and Ireland and have evidenced the rise of the ST131 as well as antibiotic resistance [19, 20]. More recently, a broadly stable population structure of bloodstream infection strains based on serotypes was observed between 2008 and 2018 in Oxfordshire (UK) [21].

In this context, we have thoroughly studied, using whole genome sequencing, two collections of *E*. *coli* strains responsible for bloodstream infections collected in 2005 and 2016-7 in teaching hospitals from the Paris area to evidence population structure dynamics over a 12-year period. First, we studied both collections on a global scale, including phylogroup determination, resistance, and virulence content. Second, we went deeper at the ST complex (STc) scale. Finally, we put special emphasis on the description of within STc population structure evolution, as few data are available at this level of granularity [20, 22, 23]. Through this analysis, we obtained a detailed picture of the evolution of the population involved in bloodstream infections as well as elements that could explain this phenomenon.

## Methods

### Clinical studies and strain collections

The strains studied here were collected from blood cultures of hospitalized adult patients suffering *E*. *coli* bloodstream infections, enrolled in two large multicentric observational studies, Colibafi (Ethics Committee CPP Hôpital Saint Louis, Paris, France: number 2006-4) and Septicoli (ClinicalTrials.gov: identifier NCT02890901) conducted in 2005 and 2016-7, respectively, in Paris and its close suburbs. Only patients previously included in the study and patients receiving vasopressors before the onset of bloodstream infection were excluded. Both studies aim was to the identify risk factors of mortality at day 28 in *E*. *coli* bloodstream infections [3, 6]. To limit epidemiologic biases, we focused only on strains isolated in teaching hospitals located in Paris and its close suburbs (which accounted for 8 among the 15 hospitals included in the Colibafi study for a total of 3,900 adult acute care beds); the other hospitals were in the rest of France. For the Septicoli study, all 7 hospitals included were in the Paris area (5,800 acute care beds), and 4 were common between the two studies (i.e., 2900 acute care beds). All the Paris area teaching hospitals belong to the same institution, the “Assistance Publique-Hôpitaux de Paris” network (www.aphp.fr), which accounts for a total of 13,000 adult acute care beds with a homogenous management for most bacterial infections. These hospitals receive each year 10 millions of patients. As the Paris area is home to 12 million people (18% of the French population), our study can be considered as representative of the French capital, characterized by a high density and multinational exchanges.

For each patient suffering from *E*. *coli* bloodstream infection, the first *E*. *coli* cultured from blood cultures was analyzed. A total of 912 strains from 912 patients (one bloodstream strain per patient) were thus studied, 367/374 strains (the remaining 7 failed to grow) from the Colibafi collection, hereinafter referred to as “2005” [3] and 545 strains from the Septicoli collection, hereinafter called “2016-7” [6]. The mean age of patients is 64.1 and 68.5 years for the 2005 and 2016-7 collections, respectively, with a female to male ratio of 1.4 and 0.9.

### Genome sequencing

Bacterial genomes were sequenced using Illumina NextSeq technology as previously described [6]. The genomes from the 2005 collection were sequenced in the present work (Bioproject PRJEB39260, https://www.ncbi.nlm.nih.gov/bioproject/?term = PRJEB39260) [24] whereas the genomes from the 2016-7 collection were previously available (Bioproject PRJEB35745, https://www.ncbi.nlm.nih.gov/bioproject/?term = PRJEB35745) [6].

### Genome global analysis and typing

All genomes were assembled with shovill version 1.0.4 [25] using SPAdes v3.13.1 [26] and standard parameters, and then annotated with Prokka 1.14.5 [27]. Genome typing was performed as previously described including species identification, phylogrouping, multi-locus sequence type (MLST) determination according to the Warwick scheme and in silico serotyping [28–30]. STcs were defined as single or double locus variant based on the MLST data of the Warwick scheme using PHYLOViZ [31] in congruence with the core genome based phylogeny (see below). Resistance genes were scanned with Resfinder v3.2 [32]. Virulome was analyzed with a custom database consisting of VirulenceFinder [33], VFDB [34], and specific genes from extra-intestinal *E*. *coli*, as previously described in the Table S2 of [30]. We also searched for point mutation responsible for betalactam (*ampC* promoter) and fluroquinolone (*gyrA/B*, *parC/E*) resistance [35]. Virulence genes were classified into 6 families: invasin, protectin, toxin, adhesin, iron acquisition, and miscellaneous [30]. Contig locations, i.e., plasmid or chromosome, were predicted using PlaScope [36]. We search for plasmid replicons using PlasmidFinder database, as previously described [30, 37]. Integrons were searched using Integronfinder with standard parameter [38].

A pangenome was computed using Roary v3.12 with default parameters [39]. Then, a phylogeny based on a core genome alignment [40] was performed with Iqtree v1.6.12 following the protocol described in [41]. Only the top 10 STcs of each collection were further studied.

### Resistance phenotype prediction

Beta-lactamase types and alleles were controlled based on the Beta-Lactamase DataBase [42] and the Bacterial Antimicrobial Resistance Reference Gene Database when necessary [43]. From these results, we predicted phenotypic resistance to clinically relevant antibiotics of 4 classes: (i) betalactams including ampicillin (AMP), piperacillin/tazobactam (TZP), cefotaxime/ceftazidime (CTX/CAZ), cefepime (FEP), carbapenems (CARB); (ii) fluoroquinolones (FQ); (iii) aminoglycosides including gentamicin (GEN) and amikacin (AMK); and (iv) cotrimoxazole (SXT), as previously described [44]. The predicted phenotypes arising from presence of genes or mutations are described in Additional file 1: Table S1. This approach was validated on the 2016-7 collection where phenotypic antibiograms (susceptible/resistant) were available as a correlation of 96.1% was found (data not shown).

### Within STc analysis

From the phylogenetic tree, we computed patristic distances (the sum of branch lengths in the path between two genomes in the phylogenetic tree) between all strains among the main 11 STcs, as described previously [41], using the function cophenetic from R package “ape” [45]. We also computed the genome fluidity [46] for all pairs of strains from the pangenome considering only variables gene families (i.e., present in less than 95% of strains). This ratio ranges from 0 to 1: the higher the ratio is, the higher the genome fluidity is, i.e., the diversity in terms of gene composition.

Moreover, to characterize more thoroughly the five main STcs (STcs 131, 95, 73, 69, and 10), we aligned their genomes to a specific reference (EC958, UTI89, CFT073, UMN026 and K-12 genomes, respectively) using Snippy 4.4.0 with standard parameters [47]. Then, we constructed a phylogenetic tree using Iqtree v1.6.12 [40] after taking into account the recombinations using Gubbins [48] with standard parameters. These SNP-based trees were then visualized and annotated with Itols [49]. We classified strains according to subgroups/clades in the main five STcs. STc131 strains were classified among clades A, B, and C based on canonical SNPs defined previously [50]. For the STc95, we classified strains according to subgroups A, B, C, D, E, or unassigned as described in [51]. Since no unified classification schemes are currently available for the STc73, STc69, and STc10, we performed a clustering analysis using fastBAPS [52] based on the SNP-based phylogenetic tree for each STc.

### Pathogenicity island analysis

We performed a detailed analysis of the *papGII* genomic context for the STc69 and STc131 strains. First, we performed blastN alignments on the NCBI website using the contigs containing *papGII* as query to look for the closest circularized *E*. *coli* genome. Then, we extracted from these complete genomes the nucleic sequences of the PAI containing *pap* genes and aligned the reads of our strains to the corresponding reference sequence using Breseq [53]. Finally, we checked if the whole length of the PAI was covered. We also checked for the presence of expected virulence genes depending on the closest PAI we found.

### Statistical analyses

The proportion of strains among each phylogenetic group and each STc was compared between the two collections (2005 and 2016-7) using *χ*2 test. Phylogroup E and *Escherichia* clades were grouped together and only the ten most prevalent STcs of each collection were considered. For each of the main STcs, we compared patristic distances and genome fluidity distributions between both collections using Wilcoxon-Mann-Whitney tests.

We performed an ANalysis Of Variance (ANOVA) to compare the distribution of VFs among the six main functional classes (adhesion, invasion, iron acquisition, miscellaneous, protectin and toxin), the predicted plasmid length, and the number of replicons between the 2005 and 2016-7 collections at global scale. Virulence gene contents were also compared in the same way for the five STcs we focused on (STc131, STc95, STc73, STc69, and STc10). Likewise, the proportion of strains predicted to be resistant on each of the nine antibiotics were compared at the global scale and for five STcs between collections using Fisher exact tests, as well as the proportion of strains carrying complete integrons, clusters of *attC* sites lacking integron-integrases (CALIN), and integron integrase only (In0). Finally, the proportion of strains among the subgroups/clades of STc131, STc95, STc69, and STc10 were compared between collections using Fisher exact tests.

As multiple tests were performed, the *p* values were adjusted using the Benjamini and Hochberg method [54]. All statistical analyses were performed using R software (R version 3.4.2). All tests were two-sided with a 5% type I error.

## Results

### Stability of phylogroup composition and VF content is associated to an increase of antibiotic resistance level between 2005 and 2016-7 collections

**Fig. 1:**
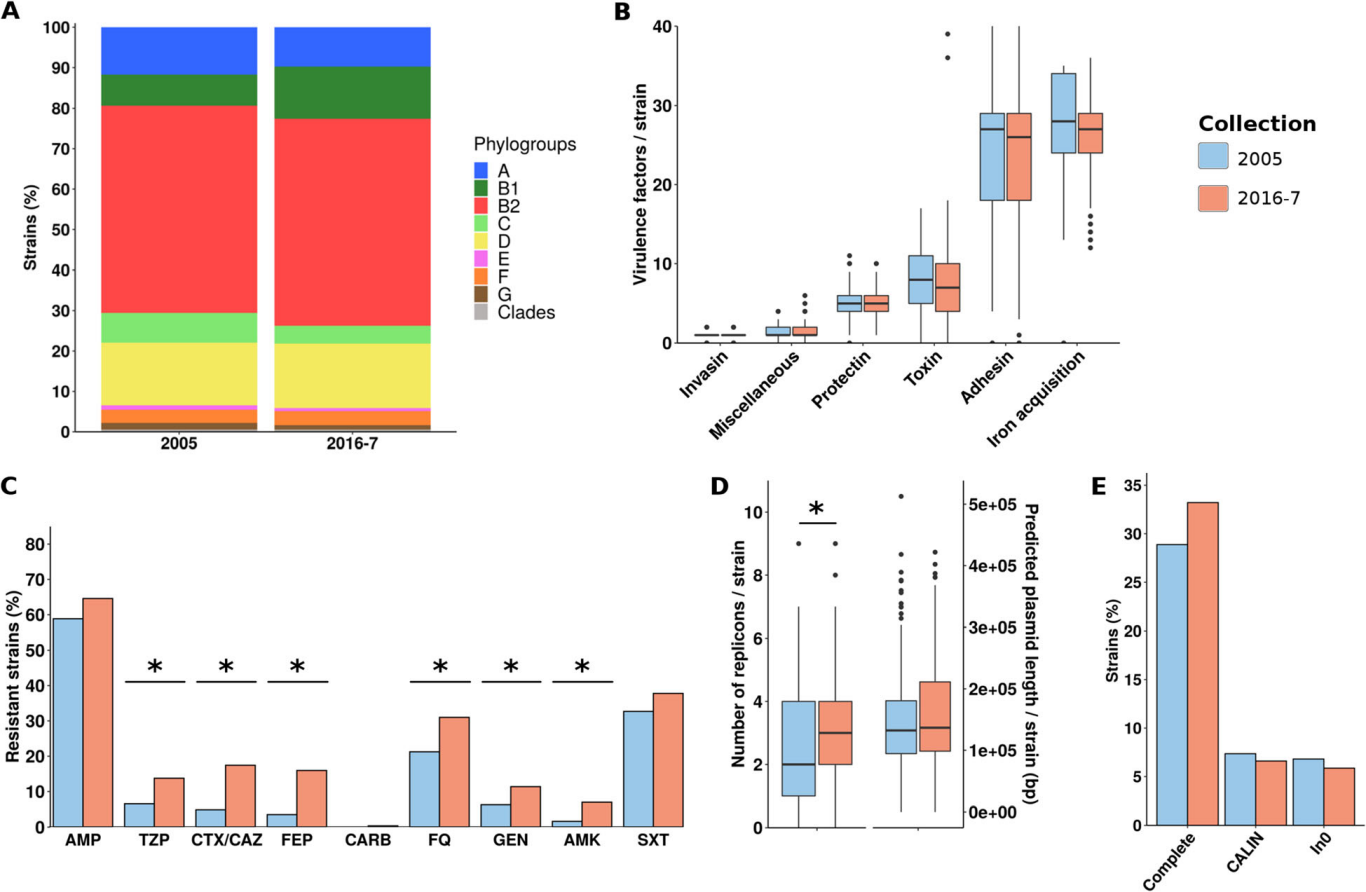
Global comparison of the 2005 and 2016-7 collections. **a** Phylogroup distribution of the strains. **b** Distribution of the number of virulence factors per strain among the six main functional classes of virulence. **c** Bar chart of predicted phenotypes of the strains. The results are presented as percentage of resistant strains for nine antibiotics of clinical importance. **d** Distribution of the number of replicons per strain and the plasmid sequence length predicted by PlaScope [36]. **e** Bar chart of the number of strains carrying complete integron, CALIN (clusters of *attC* sites lacking integron-integrases), and In0 (integron integrase only). Significant differences are highlighted by asterisks. AMP, ampicillin; TZP, piperacillin/tazobactam; CTX/CAZ, cefotaxime/ceftazidime; FEP, cefepime; CARB, carbapenems; FQ, fluoroquinolones; GEN, gentamicin; AMK, amikacin; SXT, cotrimoxazole

We sequenced and analyzed the genomes of 367 and 545 strains of *E*. *coli/Escherichia* clades responsible for bloodstream infections in adults in 2005 and 2016-7, respectively (Additional file 2: Table S2). The pangenome of both collection genomes (912 strains) is composed of 53,048 genes, with a core genome of 2269 genes that we used to build a phylogenetic tree (Additional file 3: Figure S1). We conducted a global comparison at the phylogroup level, considering the whole 912 strains (Fig. 1a, additional file 4: Table S3). Only two and three strains (0.5%) were identified as *Escherichia* clades in the 2005 and 2016-7 collections, respectively, confirming the minor role played by these clades in human [55]. *E*. *coli* strains mainly belong to phylogroup B2 (51.2% in both collection) and to a lesser extent to phylogroup D (15.5% and 16%). Then, in the 2005 collection, phylogroup A ranked third (11.7%), followed by B1 (7.6%) and C (7.4%) phylogroups, whereas in the 2016-7 collection phylogroup, B1 ranked third (12.8%), followed by A (9.7%) and C (4.4%) phylogroups. However, these differences were not significant after multiple testing corrections even when taking into account the main portals of entry (i.e., urinary or digestive) of bloodstream infections (Additional file 4: Table S3). In terms of number of virulence genes classified in main functional categories, we observed no significant differences either (Fig. 1b). In contrast, and as expected, when looking at the predicted resistance phenotype, strains from the 2016-7 collection were more often resistant to nearly all antibiotic families than the 2005 ones (Fig. 1c). For example, predicted resistance increased from 4.9 to 17.4% for cefotaxime/ceftazidime, from 21.5 to 31% for fluoroquinolones and 1.6 to 7% for amikacin. In terms of resistance determinants, we found an increase in the number of oxacillinase- and ESBL-coding genes conferring resistance to wide spectrum betalactams, *qnr*, *aac*(6’)-Ib-cr, and gyrase mutations conferring resistance to fluoroquinolones, and *aac*(3)-II/*aac*(3)-IV and *aac*(6’)-Ib-cr genes conferring resistance to aminoglycosides (Additional file 3: Figure S2). At the phylogroup level, we observed a significant increase in resistance to piperacillin/tazobactam, cefotaxime/ceftazidime, cefepime, fluoroquinolones, gentamicin, amikacin, and cotrimoxazole, however only for B2 strains (data not shown). The number of distinct replicons per strain was higher in the 2016-7 collection (mean = 2.86, SD = 1.77) than in the 2005 collection (mean = 2.51, SD = 1.66) (*p* < 0.01). We also observed an increasing trend in the plasmid sequence length predicted by PlaScope [36] in 2016-7 (mean = 148,441 bp, SD = 89,323 bp) compared to 2005 (mean = 137,506 bp, SD = 85,528 bp), although not significantly (*p* = 0.07) (Fig. 1d). We did not find any significant enrichment in integron-related sequences (Fig. 1e).

### The top 10 STcs are similar between the two collections but differ in frequency and in global genetic structure

In a second step, we compared the distribution of the top 10 STcs in both collections, corresponding to a total of 11 STcs (Table 1). These 10 STcs represent 73.5% and 73% of the strains in the 2005 and 2016-7 collections, respectively. Significant variations were observed between collections, notably the increase of the STc131, from 5.7 to 15%, and the decrease of the STc95, from 15.5 to 7%. We also noticed a slight increase of the STc69, from 8.7 to 11.6%, however not statistically significant, and which has not changed its ranking over the years. Conversely, the STc58, which corresponds to the CC87 according to the Pasteur Institute multilocus sequence typing (MLST) scheme [56], ranked 9th in 2005 (2.4%) but increased to 5.3% and to the 6th place in 2016-7.

**Table 1:** Distribution of the main STcs in *Escherichia coli* strains from the 2005 and 2016-7 collections

STc^**a**^ (Phylogroup)	Number of strains (%)		***p value***^**b**^
	2005	2016-7	
STc131 (B2)	21 (5.72)	82 (15.05)	< 0.001
STc73 (B2)	51 (13.9)	68 (12.48)	0.586
STc69 (D)	32 (8.72)	63 (11.56)	0.371
STc95 (B2)	57 (15.53)	38 (6.97)	< 0.001
STc10 (A)	28 (7.63)	33 (6.06)	0.482
STc58 (B1)	9 (2.45)	29 (5.32)	0.123
STc88 (C)	25 (6.81)	23 (4.22)	0.235
STc14 (B2)	17 (4.63)	23 (4.22)	0.766
STc12 (B2)	17 (4.63)	18 (3.3)	0.480
STc141 (B2)	5 (1.36)	13 (2.39)	0.480
STc127 (B2)	8 (2.18)	8 (1.47)	0.516

^a^Only the top 10 STcs of each collection are considered

^b^Benjamini-Hochberg correction

**Fig. 2:**
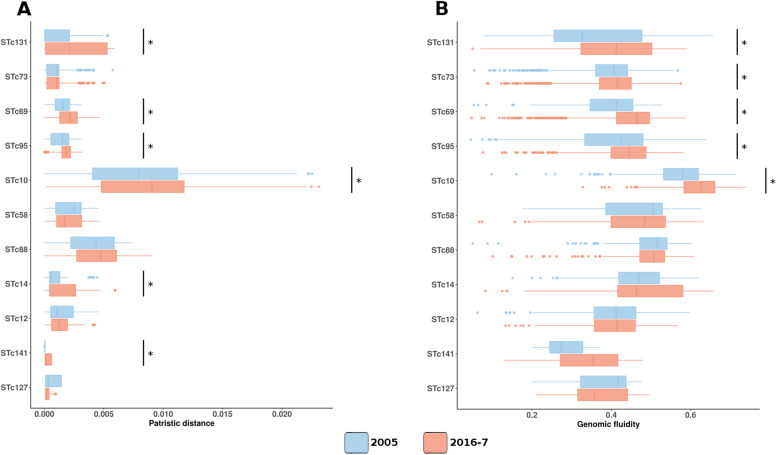
**a** Distribution of the patristic distances between all strains of a given STc in a given collection. **b** Distribution of the genome fluidity between all strains of a given STc in a given collection. Significant differences are highlighted by asterisks (Benjamini-Hochberg corrected *p* value < 0.05)

To get a more detailed picture of the evolution of these 11 STcs between 2005 and 2016-7, we computed both patristic distances and genome fluidity between strains of the same STc in a given collection (Fig. 2). The first metric reflects the genetic distance at the nucleotide level whereas the second one indicates the diversity in terms of gene content. Comparison between STcs, whatever the date of isolation of the strains, showed that STc10 behaved differently from other STcs as it had a greater genetic diversity with both metrics than the other STcs (*p* < 0.05). When comparing the evolution of diversity overtime between the two collections, we observed an increase in both patristic distances and genome fluidity, especially for STc131, STc69, STc95, and STc10.

### Fine scale analysis of the big four extra-intestinal pathogenic *E*. *coli* (ExPEC) STcs reveals highly dynamic population structure

To document more thoroughly the evolution of within STc population structure, we focused on the four main STcs, namely STc131, STc95, STc73, and STc69. These four STcs, which belong to B2 and D phylogroups, encompass typical ExPEC strains and are currently the main ones involved in bloodstream infections worldwide [57].

#### STc131

**Fig. 3:**
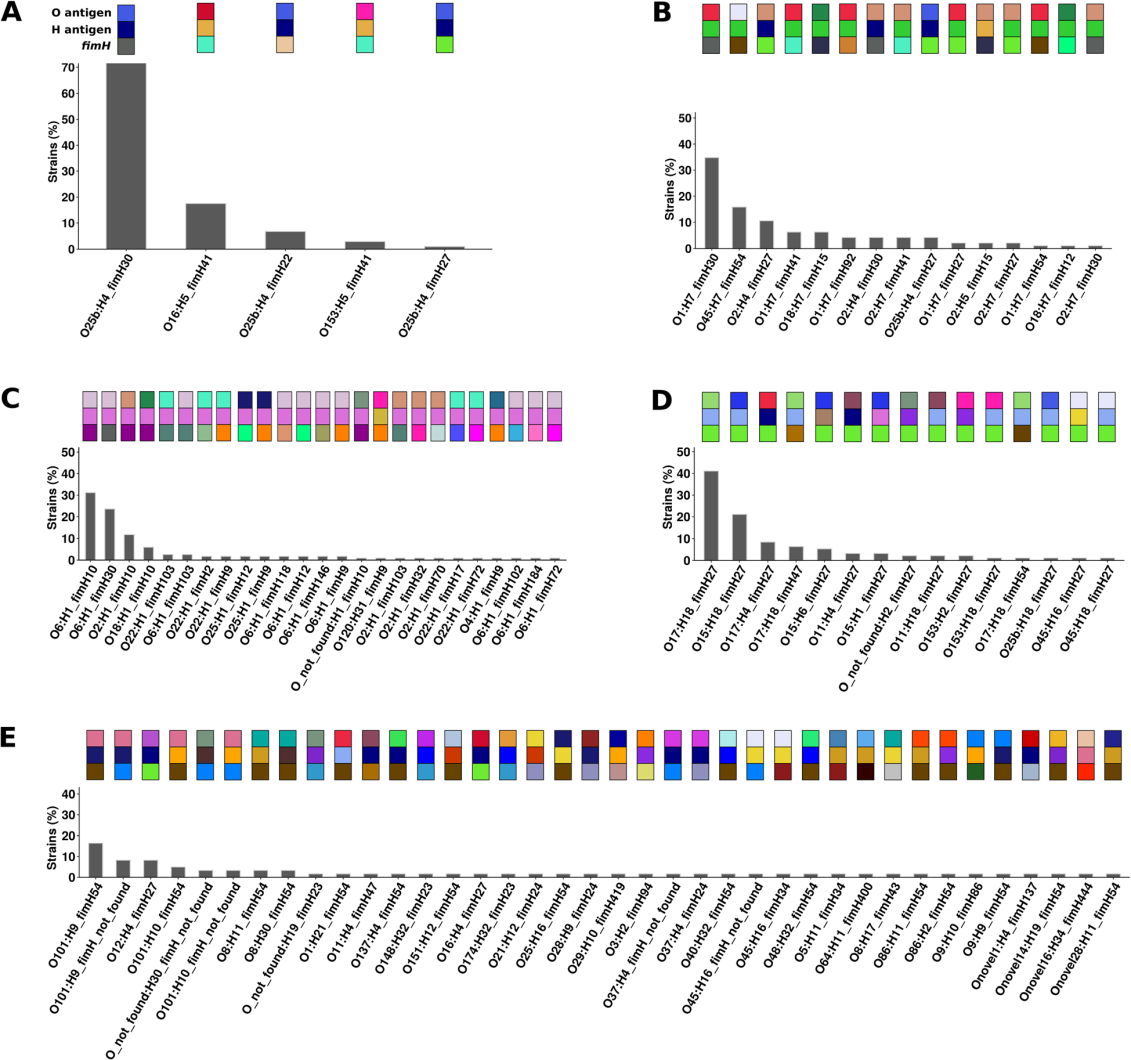
Distribution of the combinations O:H/*fimH* among the big four STcs and the STc10. **a** STc131. **b** STc95. **c** STc73. **d** STc69. **e** STc10. To have a rapid overview, the combinations O:H/*fimH* are also schematically represented by colored squares at the top of each bar graph

**Fig. 4:**
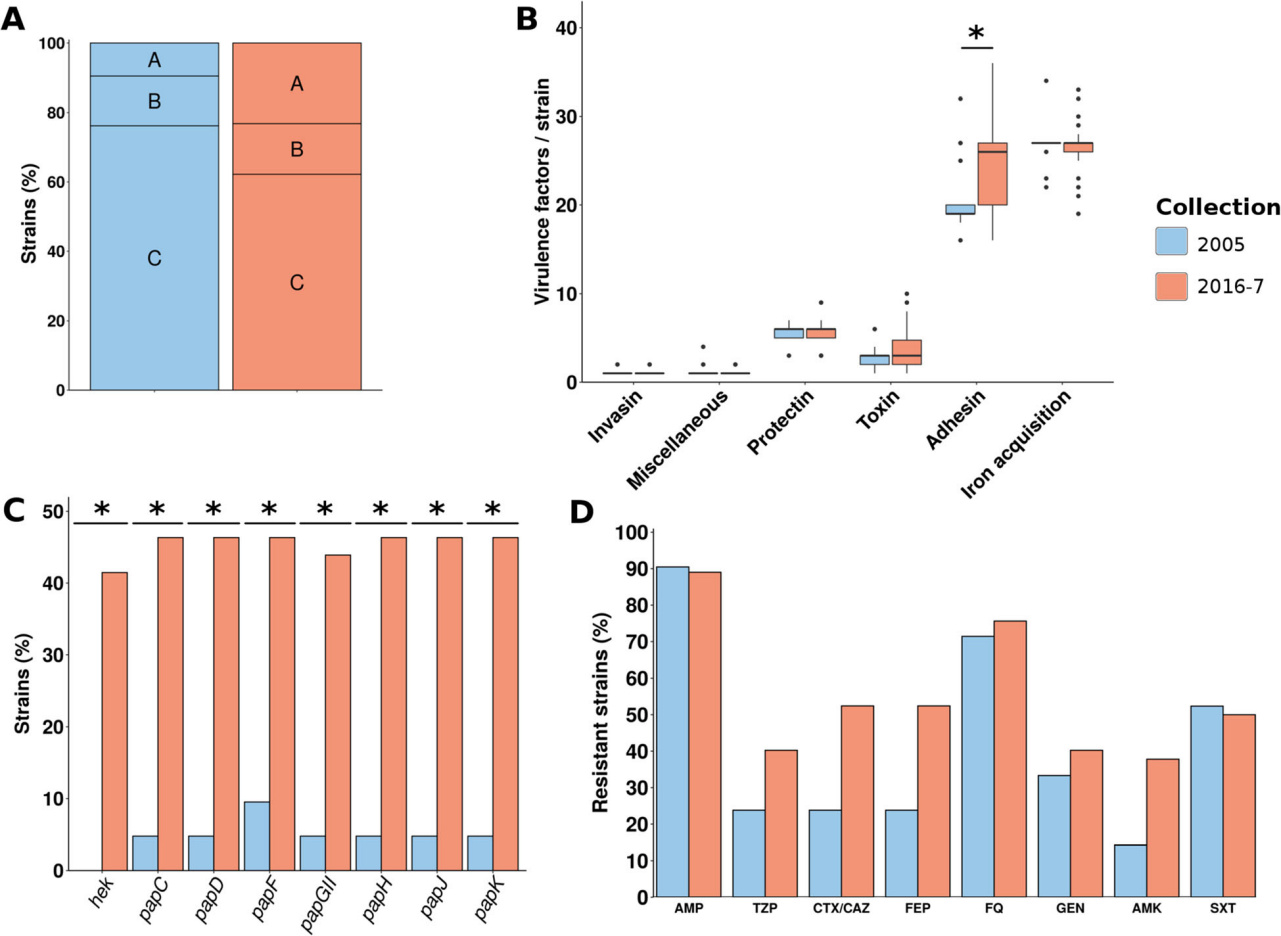
Comparison of STc131 strains in the 2005 and 2016-7 collections. **a** Distribution of strains in the three clades of STc131 described by Ben Zakour et al. [50]. **b** Distribution of the number of virulence factors per strain among the six main functional classes of virulence. **c** Distribution of the adhesins in both collections. Only adhesins with a significant difference between 2005 and 2016-7 are presented (Benjamini-Hochberg corrected *p* value < 0.05). **d** Predicted phenotypes of the strains. The results are presented as percentage of resistant strains for eight antibiotics of clinical importance (no carbapenem-resistant strain has been found). Significant differences are highlighted by asterisks. AMP, ampicillin; TZP, piperacillin/tazobactam; CTX/CAZ, cefotaxime/ceftazidime; FEP, cefepime; FQ, fluoroquinolones; GEN, gentamicin; AMK, amikacin; SXT, cotrimoxazole

The STc131, from phylogroup B2, is characterized by a stepwise diversification with two serotypes (O16:H5 and O25b:H4), three clades (A, B, and C) and three *fimH* alleles (41, 22 and 30, respectively), all correlated [50] (Fig. 3a, additional file 3: Figure S3). In our data set, we observed a slight decline of the clade C (O25b:H4) in 2016-7 balanced by a slight increase in clade A (O16:H5), which corresponds to the more diverged clade (Fig. 4a, additional file 3: Figure S3). These changes are reflected by an increase of the STc genetic diversity (Fig. 2). Moreover, clade C strains from the 2005 collection mainly belong to subclade C1, whereas in 2016-7, they are predominantly from subclade C2, which is frequently resistant to both fluoroquinolones and third generation cephalosporins (Additional file 3: Figure S3). The C1-M27 clade harboring CTX-M-27 coding gene previously reported [58] is emerging in the 2016-7 collection. In 2016-7, we also observed the emergence of a cluster of strains in the clade B with the *fimH*30 allele, as previously described [59].

In terms of virulence, only adhesins were significantly increased (adjusted *p* value < 0.01), and in particular the *hek* and *pap* genes (*papC*, *papD*, *papF*, *papGII*, *papH*, *papJ*, and *papK*) which rose from 0 and 9.5 to 41.5 and 46.3%, respectively. All of these VFs were predicted to be located on the strains’ chromosomes, and the *pap* genes were always co-localized on the same genomic region. They were distributed mostly in genomes from the subclade C2, but also in three closely related strains from clade A having the entire *bla*CTX-M-27 coding gene and the GyrA S83L mutation. We further analyzed the genetic context of these *pap* genes as they are usually found on pathogenicity islands (PAIs). In subclade C2, we were able to link the *pap* genes to at least three main PAIs in accordance both with the phylogeny and the virulence gene content of the strains (Additional file 5: Table S4): the PAI SCU-387, the PAI RHBSTW-00440 which is probably a shortened version of the PAI Ecol_AZ146 (Additional file 3: Figure S4). The latter includes also *hly* and *cnf1* genes. In the clade A strains carrying *papGII*, we found a homology with the PAI WP5-S18-ESBL-09 containing also *hly* and *cnf1* genes. This PAI presents a strong similarity with the PAI Ecol_AZ146 (Additional file 3: Figure S4). Of interest, most of these PAIs were inserted next to the tRNA-PheU and few strains presented alternative integration sites (tRNA-PheV or between *glnH* and *glnP* genes) (Additional file 5: Table S4). Taken together, these data suggest multiple transfer events of a PAI containing the *pap* gene cluster in the STc131. Besides, only five O:H/*fimH* combinations were evidenced (Fig. 3a). In terms of resistance, we observed a tendency towards more resistance including TZP, CTX/CAZ, FEP, and AMK, however not statistically significant after correction.

In summary, we observed an increase in the number of strains from clade A (O16:H5, *fimH*41) within the STc131 including closely related strains isolated in 2016-7 carrying the same PAI and resistance genes. The proportion of clade B remains stable overtime, but in 2016-7, we noticed the emergence of uncommon strains exhibiting the *fimH*30 allele. The clade C decreased slightly and we observed, except for the emerging C1-M27 clade, a switch to the C2 sub-clade with *bla*CTX-M-15 and GyrA S83L/D87N mutations as well as a high frequency of the *papGII* gene in related PAIs. Thus, the rise of the STc131 overtime comes both with the emergence of specific clones and the acquisition of virulence factors (*papGII*) through independent genetic events and specific resistance determinants, while maintaining a very low antigenic diversity.

#### STc95

**Fig. 5:**
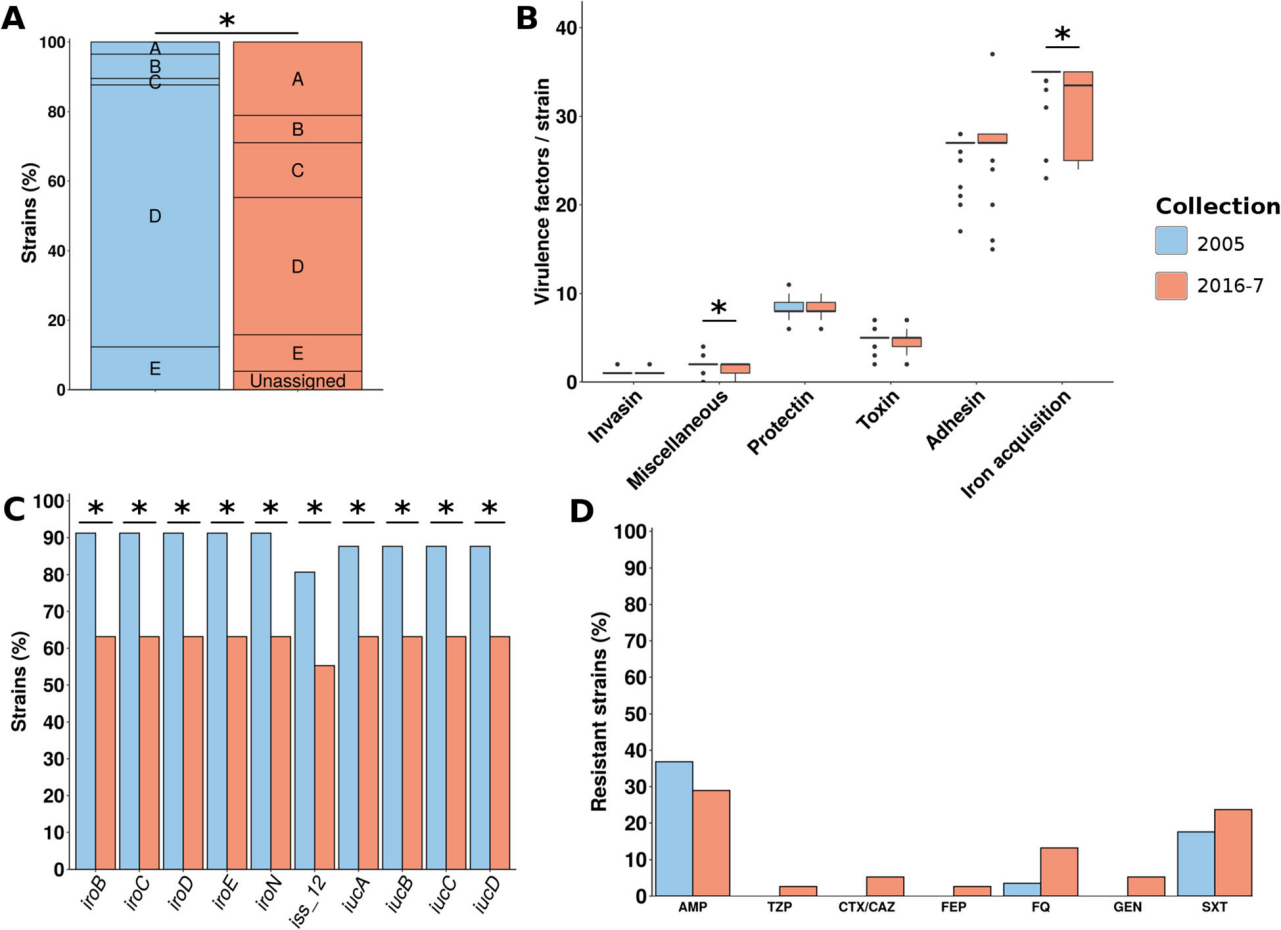
Comparison of STc95 strains in the 2005 and 2016-7 collections. **a** Bar chart of the distribution of strains in the five subgroups of STc95 described by Gordon et al. [51]. **b** Distribution of the number of virulence factors per strain among the six main functional classes of virulence. **c** Distribution of the iron acquisition related genes in both collections. Only virulence factors with a significant difference between 2005 and 2016-7 are presented. **d** Predicted phenotypes of the strains. The results are presented as percentage of resistant strains for seven antibiotics of clinical importance (no strain resistant to carbapenems and amikacin has been found). Significant differences are highlighted by asterisks. AMP, ampicillin; TZP, piperacillin/tazobactam; CTX/CAZ, cefotaxime/ceftazidime; FEP, cefepime; FQ, fluoroquinolones; GEN, gentamicin; SXT, cotrimoxazole

The STc95, also from the B2 phylogroup, has diversified rapidly leading to a star-like phylogeny with five subgroups (A to E) and serotypes specific to subgroups (O18:H7 and B, O45:H7 and D) or shared between subgroups (O1:H7 and A, C, D) [51]. Between the two collections, a major change in subgroup composition was observed, with a decrease in subgroup D strains and an increase in subgroup A strains, both significant statistically (adjusted *p* values < 0.01 and = 0.03, respectively), with the emergence of a O1:H7 *fimH*41 clone (Fig. 5, additional file 3: Figure S5). Of note, we also observed an increase in subgroup C due to the emergence of O25b:H4/O1:H7 *fimH*27 strains. The strains from the 2016-7 collection carry less iron acquisition related VFs (adjusted *p* value < 0.01), especially *iroB*, *C*, *D*, *E*, *N*, *iss_12* and *iucA*, *B*, *C*, *D*. These genes are almost exclusively found on plasmidic contigs, *iro* genes and *iss_12* being almost always co-localized on the same contig, and *iuc* genes on another. We also found less VFs of the miscellaneous class (adjusted *p* value = 0.04), partly due to the less frequent presence of *etsC* gene encoding a putative type I secretion outer membrane protein. As these iron acquisition and miscellaneous genes are typically carried by the pS88 plasmid (accession number: CU928146), we searched for its presence in our strains using blastN alignments. The plasmid was detected in nearly all strains, including subgroup D, but was not found in the emerging subgroup A. The antigenic diversity is constrained with 15 O:H/*fimH* combinations (Fig. 3b). Finally, we observed a tendency toward greater antibiotic resistance (TZP, CTX/CAZ, FEP, FQ, GEN, SXT), but not statistically significant as in STc131.

In summary, while STc95 strains were the most numerous in the 2005 collection, their decrease in 2016-7 is associated with a more balanced population structure. This is linked to the emergence of subgroups C and A, the latter lacking the pS88-related genes implicated in iron acquisition.

#### STcs 73 and 69

As no consensus nomenclature on population genetic structure was available for the STcs 73 [20, 60] and 69 [20, 60, 61] (B2 and D phylogroups, respectively), we merged the strains of the two collections and used fastbaps [52] to have an overview of the strain diversity within these STcs. Concerning the STc73, the tree showed a polytomy (i.e., a multifurcation) with low values of patristic distances (Fig. 2, additional file 3: Figure S6), indicating very few diversification. Fastbaps identified 6 subgroups, some of them exhibiting specific ST (subgroup A: ST104) or serogroup (subgroup B: O22/O25, subgroup D: O2, subgroup F: O18), in global accordance with [20] (Additional file 3: Figure S6). Moreover, a total of 25 O:H/*fimH* combinations were observed (Fig. 3c). We did not evidence any significant differences in terms of subgroups between the collection origins (Additional file 3: Figure S6). In terms of virulence and resistance, we did not identify any significant difference. All these elements point to the absence of emerging clones over the years but a relatively high antigenic diversity.

**Fig. 6:**
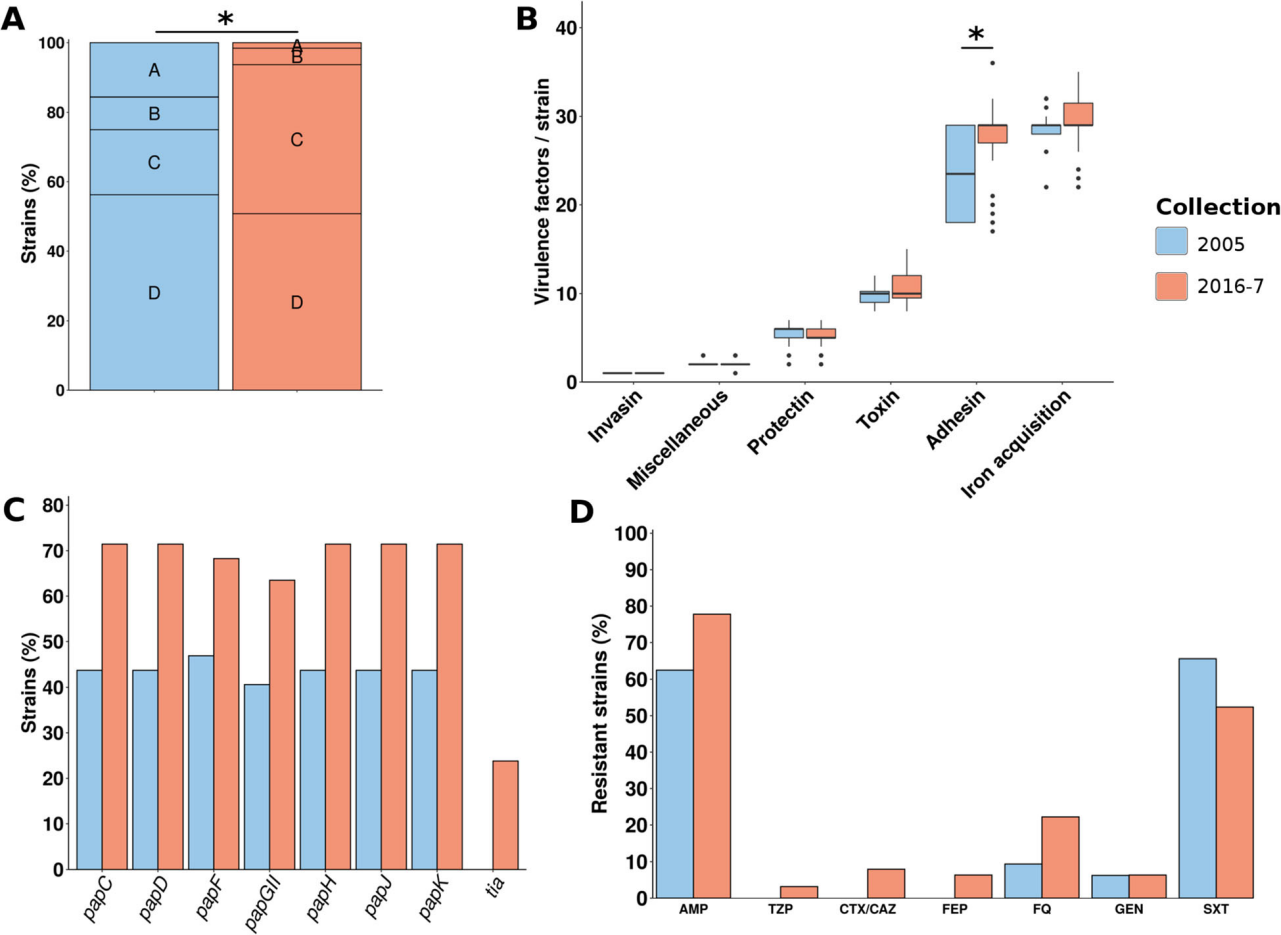
Comparison of STc69 strains in 2005 and 2016-7 collections. **a** Bar chart of the distribution of strains in the four subgroups of STc69 defined by fastbaps [52]. **b** Distribution of the number of virulence factors per strain among the six main functional classes of virulence. **c** Distribution of the adhesins in both collections. Only virulence factors with the most significant differences (i.e., significant before multiple test correction) between 2005 and 2016-7 are presented. **d** Predicted phenotypes of the strains. The results are presented as percentage of resistant strains for seven antibiotics of clinical importance (no strain resistant to carbapenems and amikacin has been found). Significant differences are highlighted by asterisks. AMP, ampicillin; TZP, piperacillin/tazobactam; CTX/CAZ, cefotaxime/ceftazidime; FEP, cefepime; FQ, fluoroquinolones; CARB, carbapenems; GEN, gentamicin; SXT, cotrimoxazole

Concerning the STc69, the SNP-based phylogenetic tree reconstructed from the strains of both collections delineated two main groups further delineated in four subgroups by fastbaps (namely A, B, C, and D) (Additional file 3: Figure S7), in accordance with [20, 60]. The subgroup B corresponds to ST106 strains. Almost all the strains exhibit a *fimH*27 allele (*n* = 88/95). We found a decrease of subgroup A strains and an increase of subgroup C strains in 2016-7 compared to 2005 (adjusted *p* value = 0.047) (Fig. 6). At the STc scale, the strains of the 2016-7 collection have a higher number of adhesins than the strains from 2005, but not statistically significant. As in the STc131, these additional adhesins are part of the *pap* gene cluster (*papC*, *papD*, *papF*, *papGII*, *papH*, *papJ*, *papK*, and *tia*). This increase in adhesins is partly linked to a clone of 14 strains of the 2016-7 collection in subgroup C. These strains exhibit an O15:H18 serotype and carry for almost all the *sul1*, *sul2*, *dfrA*, *papGII* genes, and a truncated *hlyC* gene (Additional file 3: Figure S7). We also observed the emergence of a O117:H4 *fimH*27 clone in the D subgroup exhibiting the *pap* genes. The analysis of the genetic context of *papGII* showed at least two different paths of acquisition of the adhesins (Additional file 5: Table S4). On one hand, in subgroup D, we found the typical PAI of STc69 archetypal strain UMN026 (NC_011751.1) inserted in tRNA-PheU, sometimes partly deleted, which usually carries *pap* genes, *iha*, *sat*, *iutA*, *iucA*, and the capsule coding genes *kpsMDE* (Additional file 3: Figure S8). This PAI is closely related to the ATCC25922 PAI found in the STc131 at the difference of the *hly* genes that are absent (Additional file 3: Figure S4). On the other hand, in subgroup C, we found a PAI carrying *papGII*, *tia*, *ireA* and inserted in tRNA-PheU (Additional file 3: Figure S8). The level of antigenic diversity is similar to the STc95 one with 15 O:H/*fimH* combinations (Fig. 3d). Strains tend to be slightly more resistant to some antibiotics (AMP, TZP, CTX/CAZ, FEP, FQ), but not significantly.

In summary, we observed a slight increase, although not significant, in the number of STc69 strains overtime. Moreover, we found significant subgroup variations including an increase of the subgroup C, which contains an emerging clone with a O15:H18 serotype and *papGII* on an uncommon PAI. A second clone emerged in the subgroup D with a O117:H4 serotype and the archetypal PAI of ST69.

### The particular case of the STc10

In terms of prevalence, the fifth STc is the STc10, which encompasses typically commensal strains devoid of intrinsic extra-intestinal virulence [12]. We observed for this STc a high level of diversity with both patristic distance and genome fluidity metrics (Fig. 2). Three subgroups can be distinguished by fastbaps (A, B, and C) (Additional file 3: Figure S9), the subgroups A and B corresponding to the ST48 and ST10, respectively, whereas the subgroup C encompasses O101:H9/10 *fimH*54 strains. These last strains exhibit GyrA/ParC mutations and ESBL coding genes for some of them. Except subgroup C, a huge antigenic diversity was observed with 38 widespread O:H/*fimH* combinations (Fig. 3e). No difference was evidenced between the collections in terms of subgroup repartition, virulence factors, and antibiotic resistance and no clear pattern of emerging clone was observed (Additional file 3: Figure S9).

In summary, the STc10 exhibits a unique pattern of diversification with a huge antigenic diversity for two subgroups and a third subgroup encompassing a specific serotype, all present in both collections and not linked to recent emerging clones.

## Discussion

Since the last 20 years, the molecular epidemiology of *E*. *coli* has dramatically changed with the emergence of some of the current most prevalent lineages, i.e., STc131, STc69, bringing with them extensive antibiotic resistance [57]. Numerous studies focused on the diversification of specific STs as the ST131 [50, 62], ST95 [51], and ST69 [61] and gave clues on the process leading to within ST clade formation. However, to our knowledge, few studies have analyzed diversification within STc using time-series data [20, 22, 23]. Such fine scale analyses of sequential isolates overtime are mandatory to understand the evolutionary forces at play. From two epidemiologically comparable collections of *E*. *coli* strains isolated from bloodstream infections during two multicentric observational studies conducted 12 years apart in the same institution in the Paris area, we were able to describe the evolution of the bacterial population structure overtime at different levels of granularity.

The first striking result of our study is the remarkable stability in phylogroup composition and virulence gene content between the 2005 and 2016-7 collections (Fig. 1). The proportion of B2 phylogroup strains (53%) is surprisingly identical in the two collections despite an observed increase in the frequency of B2 and their VF content for commensal strains isolated in the same area during the period 1980–2010 [18]. The incidence of B2 strains in bloodstream infections is dependent on the portal of entry, urinary tract infections being associated with the higher proportion, i.e., 60% [63] (Additional file 4: Table S3). Of note, this stability of B2 phylogroup strain proportion was already observed in a collection of 34 bloodstream infections strains from urinary portal of entry isolated in the 1980s in one of the hospital of the present study, where carboxyl esterase of B_2_ type (corresponding to B2 phylogroup) strains represented 56% [64].

The fact that the trends in phylogroup distribution and VFs observed overtime in commensal strains [18] are not found for bacteremia strains can be explained if bacteremia strains are a very selected subset of commensal strains. This suggests that a specific pattern of phylogroup diversity is adapted to the bacteremic lifestyle of the strains, probably due to specific phylogroup characteristics. Such characteristics could be linked to metabolic processes, as genes involved in metabolism were found differentially represented at the phylogroup level [41]. For example, genes involved in aromatic compound degradation are negatively and positively associated with B2 and B1 phylogroup strains, respectively [41]. Metabolic functions are fundamental for adaptation to different nutritional niches [65] and survival in the face of bactericidal defense mechanisms [66]. According to the portal of entry (urinary, digestive, pulmonary) and/or the host conditions, different phylogroups could be selected due to their ability to grow in these environments. Then, there is a stable spillover of bloodstream infection causing isolates from these restricted niches.

Beyond this stability in phylogroup repartition, we observed, as already reported in commensal strains [18], a major increase in antibiotic resistance of many classes, i.e., betalactams, fluoroquinolones, and aminoglycosides. This increase in antibiotic resistance is only statistically significant in B2 strains, due mostly to the increase of the STc131, but there is nevertheless a slight tendency in all the studied STcs. It is associated with an increase in plasmid replicons, and a trend toward longer plasmid sequence per strain, in agreement with the plasmidic origin of the majority of resistance. However, integron analyses failed to identify significant differences, suggesting that such mobile genetic elements are not the main driver of this elevated antibiotic resistance.

The second striking result is the stability of the global prevalence of the 10 main STcs, which represent almost ¾ of the strains, associated with modification of prevalence for some of them. The pauci-clonality of the strains responsible for bloodstream infections has also been observed in a study from UK [20], where our defined top 10 STcs represent at least 67% of their 1509 isolates. These data contrast with the diversity of the commensal fecal ones, in which 23 STc/ST are necessary to reach 73.5% of the whole population (*n* = 206/280 strains) [18, 63]. However, among these highly prevalent STcs, we found a major switch in the B2 phylogroup corresponding to an increase of the STc131 compensated by a decrease in STc95 (Table 1). One of the main differences between these two STcs is their antibiotic resistance phenotypes, the STc131 being multi-resistant [17, 50], whereas the STc95 is susceptible to most antibiotics, possibly due to the presence of restriction modification systems precluding the gain of foreign DNA [67]. Of interest, the intrinsic extra-intestinal virulence in a mouse model of sepsis was reported similar for both STcs [51, 68]. It is also interesting to note the increase (although not significant) in STc58 (CC87), which now ranks 6th in the 2016-7 collection. This STc of the B1 phylogroup, rarely isolated in humans, originated in animals and spread to humans, carrying antibiotic resistance determinants [56]. Until now, this lineage was considered as a harmless commensal devoid of intrinsic extra-intestinal virulence [56]. No difference was observed in the VF content or in the predicted resistance between the strains of the two collections. Further epidemiological studies will be necessary to monitor this potential emerging group and identify genetic determinants that may be responsible for this increase.

**Fig. 7:**
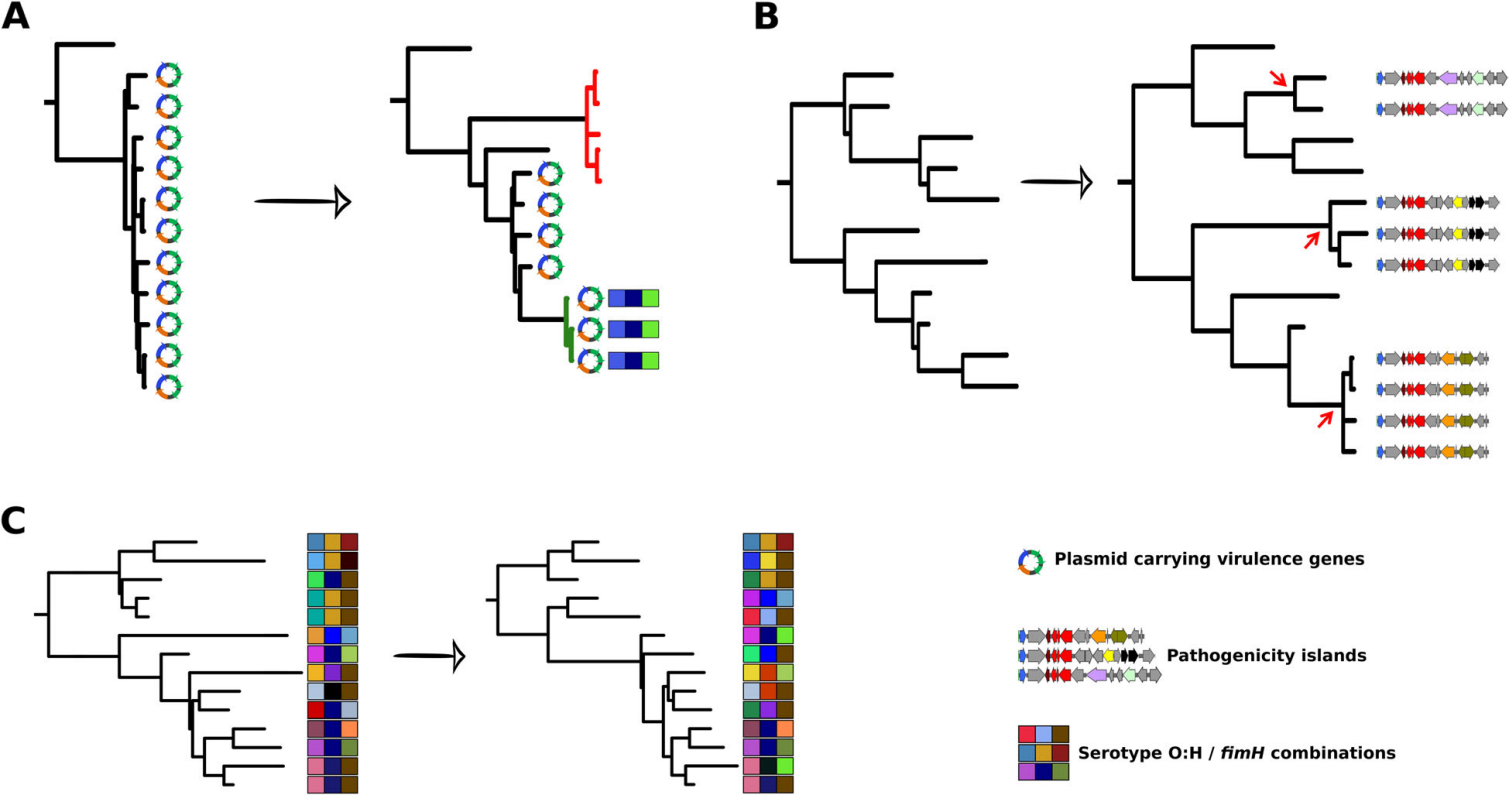
Schematic representation of the different scenarios leading to within STc dynamic. **a** Example of clonal replacement as observed in STc95. The represented plasmid corresponds to pS88 whereas the red terminal branches correspond to the emerging O1:H7 *fimH*41 subgroup A clone. The emerging O25b:H4 clone in the subgroup D is indicated by colored squares as in Fig. 3 and the branches are highlighted in green. **b** Multiple acquisitions of related PAIs associated to clonal expansion as observed in STc69 and STc131. The *pap* gene cluster with the *papGII* allele is represented in red on genetic maps. Red arrows indicate the acquisition of PAIs. **c** High antigenic diversity at a given time and overtime, as observed in STc10. This pattern corresponds probably to multiple recombination events at the main chromosomal hot spots (*rfb*, *fimH*)

The third striking result is that a change in the genetic structure of the *E*. *coli* population is underway with increased diversity overtime in several STcs (Fig. 2), which occurs regardless of changes in STc prevalence over the study period. Various evolutionary scenarios may be hypothesized:

(i) Clonal replacement. This scenario is observed in both declining (STc95) and successful (STc131) lineages. Within the STc95, clonal replacement (subgroup A and C strain increase while subgroup D strain decrease) is associated with the decrease of plasmid borne genes implicated in iron acquisition in subgroup A and apparition of a new serotype, i.e., O25b:H4 with *fimH*27, in subgroup C (Fig. 7a). Iron capture systems are the major determinants of intrinsic extra-intestinal virulence [69]. The emergence of a clone devoid of such systems could be explained by the apparition of other undetected changes, ranging from SNP(s) to gene(s)’ presence/absence linked to increasing fitness. The emergence of a clone with a serotype never observed in the STc before could argue for a potential role of this serotype, especially as it is the same than the emerging ST131 lineage (O25b:H4). However, as above, it could be linked to other factors in linkage disequilibrium with the serotype.

Within the STc131, we noticed the emergence of the clade B *fimH*30 clone, which shares some genetic features with the C clade, exhibits a reduced virulence in mice as compared to other B clade strains [59] and is not antibiotic resistant. This clone needs to be monitored to assess its fate.

(ii) Clonal replacement associated to convergent evolution. We observed an increase in the frequency of specific VFs, especially adhesins with the P-fimbriae-encoding locus exhibiting the *papGII* allele, due to multiple PAI arrivals in distinct clones, in both STc131 and STc69, and linked to clonal expansion (Fig. 7b). Such convergent evolution is a strong sign of selection [70] and has been involved frequently in the evolution of pathogenic *E*. *coli* [57, 71]. Nonetheless, according to the level of clonal divergence, this selection most likely occurred in more ancient times than the increase in frequency that took place between 2005 and 2017. Indeed, the emergence of the most recent clonal lineages within the clade C2 of the ST131 and the terminal nodes within the ST69 have been dated to the 1990s [20, 50]. In accordance with our data, *pap* gene increase was also observed in Kallonen et al. data where *papG* in ST131 raises from 8% in 2003 to 44% in 2012, as well as in a Spanish study where the authors found an increase of strains from STc131/clade C carrying *papGII* between 2006 and 2011 [72]. The importance of this event in *E*. *coli* evolution is illustrated by a recent work using a genome-wide association approach showing that, within D, F, and B2 phylogroups, repeated horizontal acquisition of diverse *papGII*-containing PAIs underlies the emergence of invasive uropathogenic lineages [60].

Furthermore, within ST131, resistance acquisition sometimes co-occurred with increase in virulence. In clade A, the emerging clone exhibiting *papGII* virulence factor also harbors the GyrA S83L mutation and *bla*CTX-M-27. When looking at the EnteroBase [73] (February 2019), among 587 *E*. *coli* ST131 carrying *bla*CTX-M-27, we find 64 O16:H5 strains among which 23 were positive for *papGII*/*hlyC*/*cnf1* and had a GyrA S83L mutation (data not shown). These strains are human ExPEC isolated worldwide after 2014. It will be interesting to see if this clone expands in the future, as observed for clade C2 that acquired the same genetic attributes [50].

Such clonal expansions in ST131 can spread all over the world as the C1-M27 clade [58, 74] whereas other have a geographical component as the recently reported clade C2 long term care facility displacement clone in Ireland [23]. Indeed, this last clone, which has chromosomal insertion of the blaCTX-M-15 disrupting the *mppA* gene, was absent in our data set (data not shown). Interestingly, the SEA-C2 clone described as highly prevalent in Southeast Asia as compared to Europe and Americas in 2015 [75] is now present in our 2016-7 collection (data not shown), indicating its worldwide diffusion.

(iii) Antigenic variation. The O polysaccharide and the H flagellin are major surface antigens [76, 77]. The fimbrial tip-positioned adhesive protein FimH is also a surface antigen [78]. The O-antigen biosynthesis gene cluster and the *fim* operon are known as the two major hotspots of recombination on the *E*. *coli* chromosome that are under diversifying selection [79]. The diversity of these antigens is variable according to the STcs [57]. Variable patterns of serotype/*fimH* allele combinations can be evidenced in the five main STcs (Fig. 3): (i) very few combinations (STc131, *n* = 5), the main one representing 70% of isolates; (ii) few combinations (STc95, *n* = 15; STc69, *n* = 15) with the main ones representing 30-40% of isolates; (iii) intermediate number of combinations (STc73, *n* = 25); and (iv) high number of combinations widely distributed (STc10, *n* = 38) with the main one representing less than 17% of isolates. This indicates that STc10 has a very specific pattern of diversification as it remains polyclonal and exhibits a huge antigenic diversity whatever the year of isolation (Fig. 7c), in line with its commensal ecology [80]. This diversity may help it resist the immune system.

Surprisingly, the STc73, which is remarkably stable in terms of frequency, is not affected by clonal replacement overtime. Moreover, this stability is also observed both in terms of resistance and virulence. Although far below the antigenic diversity observed in the STc10 (Fig. 3), the multiple combinations of O:H/*fim*H of the STc73 as well as the high frequency of *papGII* [60] could participate to its evolutionary success.

Our work has several limitations. First, only two points of sampling are available. Second, the sequencing mode by short reads impedes the circularization of plasmids and thus the detailed investigation of their role. Third, resistance analysis relies on prediction based on genes and/or mutations. However, such predictions avoid biases linked to phenotypic antibiograms and may identify low-level resistance or decreased susceptibility, which could be relevant for both fluoroquinolones and beta-lactams [81, 82]. Fourth, it is unclear how our data are representative of other areas with different population densities, patterns of human (and animal, food, and water) movement, and healthcare systems. Nevertheless, our study is epidemiologically robust as it is based on prospective and multicenter cohorts in the same institution and can be considered as representative of the French capital. Finally, the data presented here are based on genomic analyses and their clinical relevance need to be investigated. In vitro and in vivo determinations of the fitness of the emerging (and declining) clones coupled to the identification of the genetic determinants involved will help assess their role in the pathophysiology of the disease. It has to be noted that, at the highest level of integration, patient mortality is still similar between the two studies (12.6 and 9.5% for the 2005 and 2016-7 collections, respectively).

## Conclusions

Our results suggest that, depending on the level of granularity considered, contrasting patterns of evolution overtime exist. Indeed, we found a remarkable stability in terms of phylogroup distribution, a global stability in terms of main STc distribution with an increase or a decrease of some specific STcs and huge modifications within STcs with clonal interference, characterized by competition between variant clones in each STc, as well as large variations in frequency of virulence and sometimes resistance genes. This indicates a global evolutionary constraint at the phylogroup level and to a lesser extent at the STc level that is associated with a diversifying selection within STcs. The intra-STc dynamics could result from negative frequency-dependent selection, as suggested previously [20]. This selection is probably anterior to the period studied in this work and could occur in the commensal niche. Indeed, the gut is the primary habitat of *E*. *coli* [11] and the reservoir of ExPEC strains [57]. Extra-intestinal infections, especially bloodstream infections with a high level of mortality, can be considered as evolutionary dead-ends, the “virulence determinants” being in fact selected to allow a more successful gut colonization [16, 83]. This selection would drive the emergence of clones with geographic specificity, in line with the role of the environment shaping *E*. *coli* commensal microbiota [84], some of them spreading worldwide. It will be of interest to follow the fate of the actual clones in the following years. Additional worldwide epidemiologic studies are needed to determine whether these findings are generalizable in other ecological contexts.

## Supplementary Information

**Additional file 1: Table S1.** Antibiotic resistance prediction according to genes/mutations.

**Additional file 2: Table S2.** Main characteristics of the 912 strains from collections 2005 and 2016-7.

**Additional file 3: Figure S1.** Core-genome SNP based phylogenetic tree of the 912 strains from collections 2005 and 2016-7. **Figure S2.** Distribution of genes and mutations responsible for resistance to beta-lactams (A), fluroquinolones (B) and aminoglycosides (C) among strains from the 2005 and 2016-7 collections. **Figure S3.** SNP-based phylogenetic tree of STc131 strains. **Figure S4.** Genetic map of the reference PAIs found in the STc131 strains. **Figure S5.** SNP-based phylogenetic tree of STc95 strains. **Figure S6.** SNP-based phylogenetic tree of STc73 strains. **Figure S7.** SNP based phylogenetic tree of STc69 strains. **Figure S8.** Genetic map of the reference PAIs found in the STc69 strains. **Figure S9.** SNP-based phylogenetic tree of STc10 strains.

**Additional file 4: Table S3.** Phylogroup distribution among 2005 and 2016-7 collections, overall and according to urinary and digestive portal of entry.

**Additional file 5: Table S4.** Main characteristics of the studied pathogenicity islands.

## Data Availability

The whole-genome sequences of the 912 strains studied have been deposited under the Bioprojects PRJEB35745 (https://www.ncbi.nlm.nih.gov/bioproject/?term=PRJEB35745) [6] and PRJEB39260 (https://www.ncbi.nlm.nih.gov/bioproject/?term=PRJEB39260) [24].
