# Supplementary material for: Phylogroup stability contrasts with high within sequence type complex dynamics of Escherichia coli bloodstream infection isolates over a 12-year period
Source: Genome Med. 2021 May 5;13:77. doi: 10.1186/s13073-021-00892-0 (PMC8097792; doi:10.1186/s13073-021-00892-0)
Supplement: Supplementary file 3 — Additional file 3: Figure S1. Core-genome SNP based phylogenetic tree of the 912 strains from collections 2005 and 2016-7. Figure S2. Distribution of genes and mutations responsible for resistance to beta-lactams (A), fluroquinolones (B) and aminoglycosides (C) among strains from the 2005 and 2016-7 collections. Figure S3. SNP-based phylogenetic tree of STc131 strains. Figure S4. Genetic map of the reference PAIs found in the STc131 strains. Figure S5. SNP-based phylogenetic tree of STc95 strains. Figure S6. SNP-based phylogenetic tree of STc73 strains. Figure S7. SNP based phylogenetic tree of STc69 strains. Figure S8. Genetic map of the reference PAIs found in the STc69 strains. Figure S9. SNP-based phylogenetic tree of STc10 strains. [file 13073_2021_892_MOESM3_ESM.pdf]

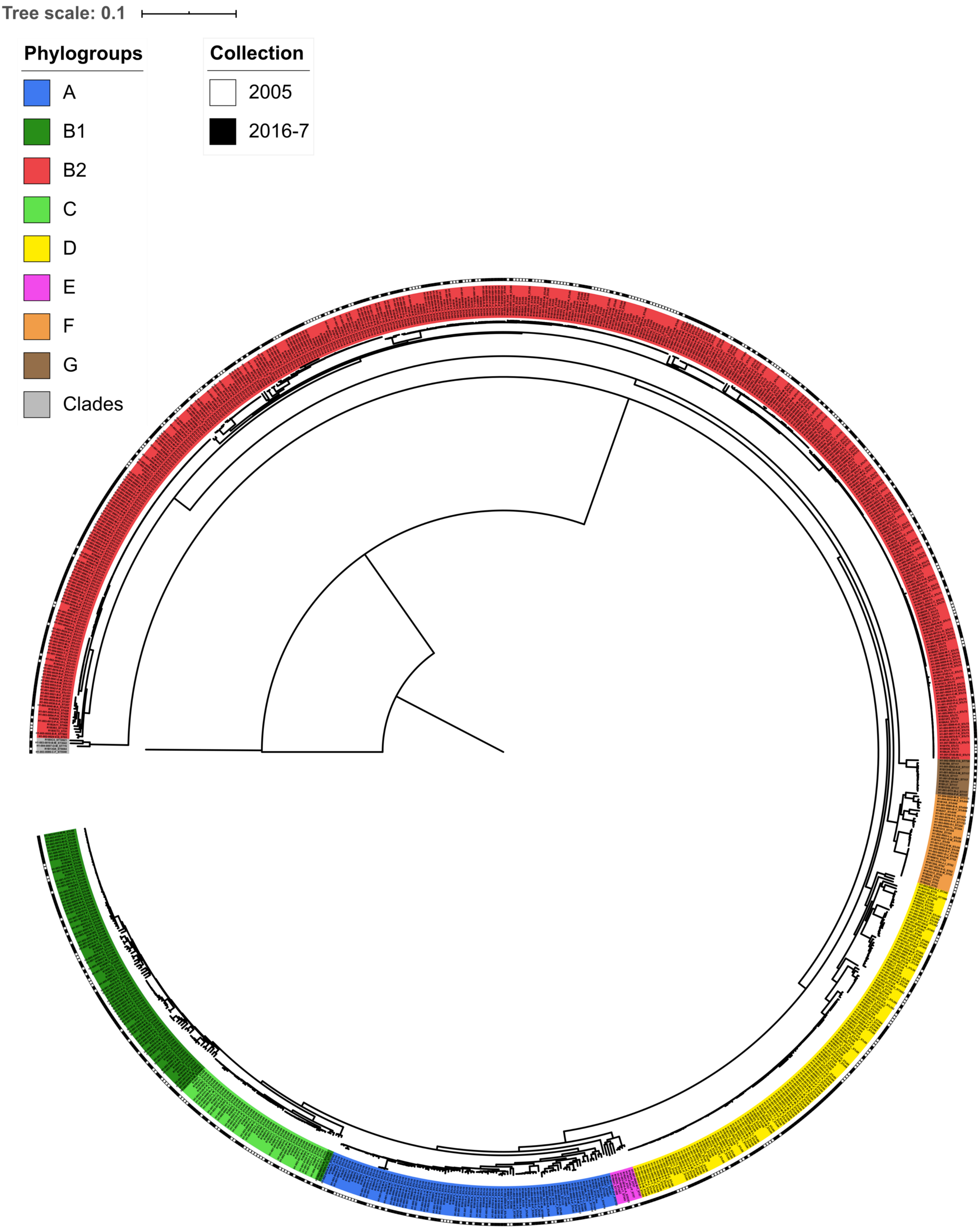

Figure S1. Core-genome SNP based phylogenetic tree of the 912 strains from collections 2005 and 2016-7. The tree is rooted on an *Escherichia* clade V strain. The eight main phylogroups are highlighted in color. On the outermost circle appear white and black squares according to the origin of the strains (*i.e.* 2005 or 2016-7, respectively). The scale represents genetic distances in nucleotide substitution per site.

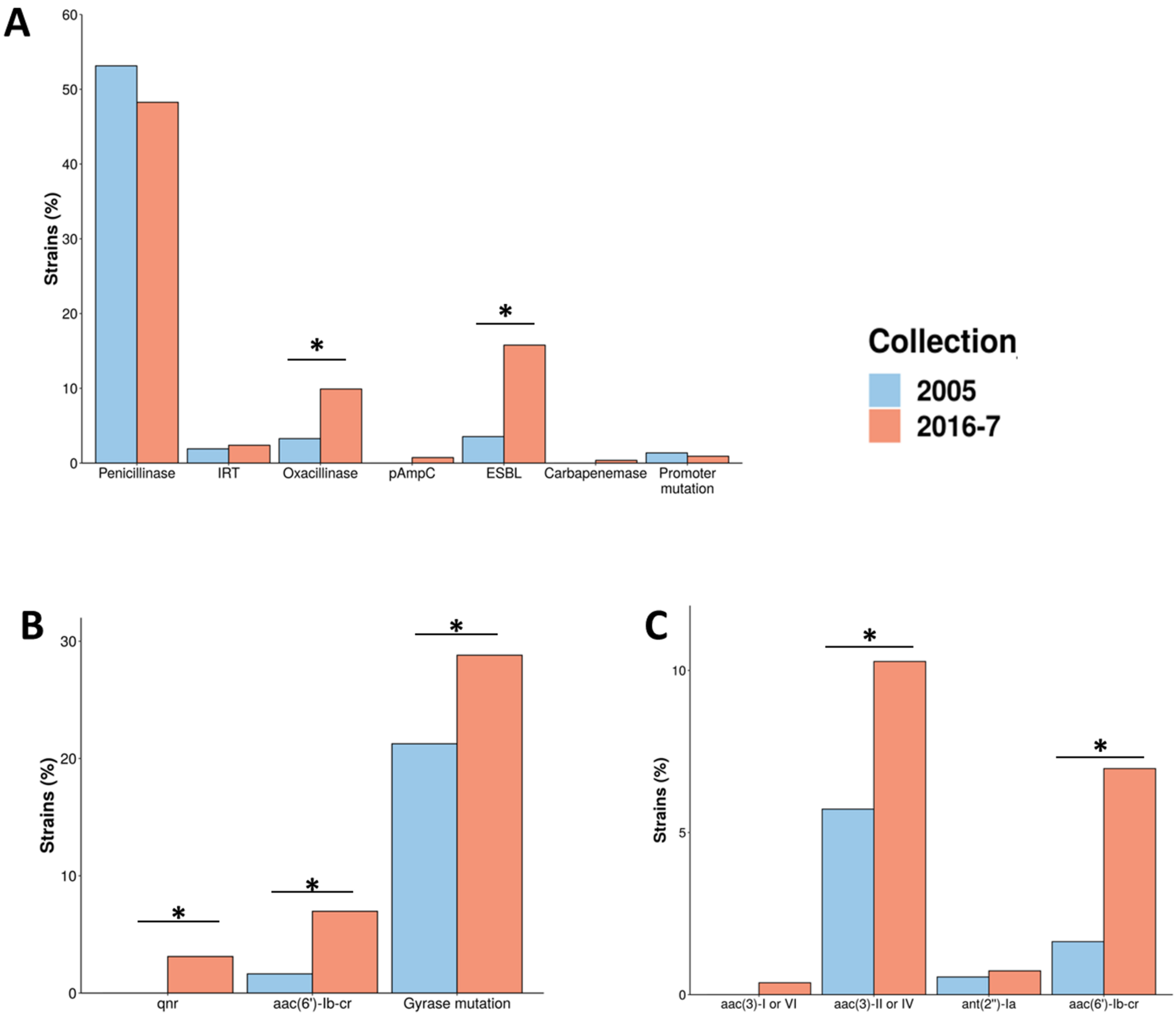

Figure S2. Distribution of genes and mutations responsible for resistance to beta-lactams (A), fluroquinolones (B) and aminoglycosides (C) among strains from the 2005 and 2016-7 collections. Betalactam resistance coding genes are grouped by phenotype. The gyrase mutations include only GyrA mutations for fluoroquinolone resistance. Significant differences are highlighted by asterisks. IRT = Inhibitor Resistant TEM; pAmpC = plasmidic ampC; ESBL = extended spectrum betalactamase.

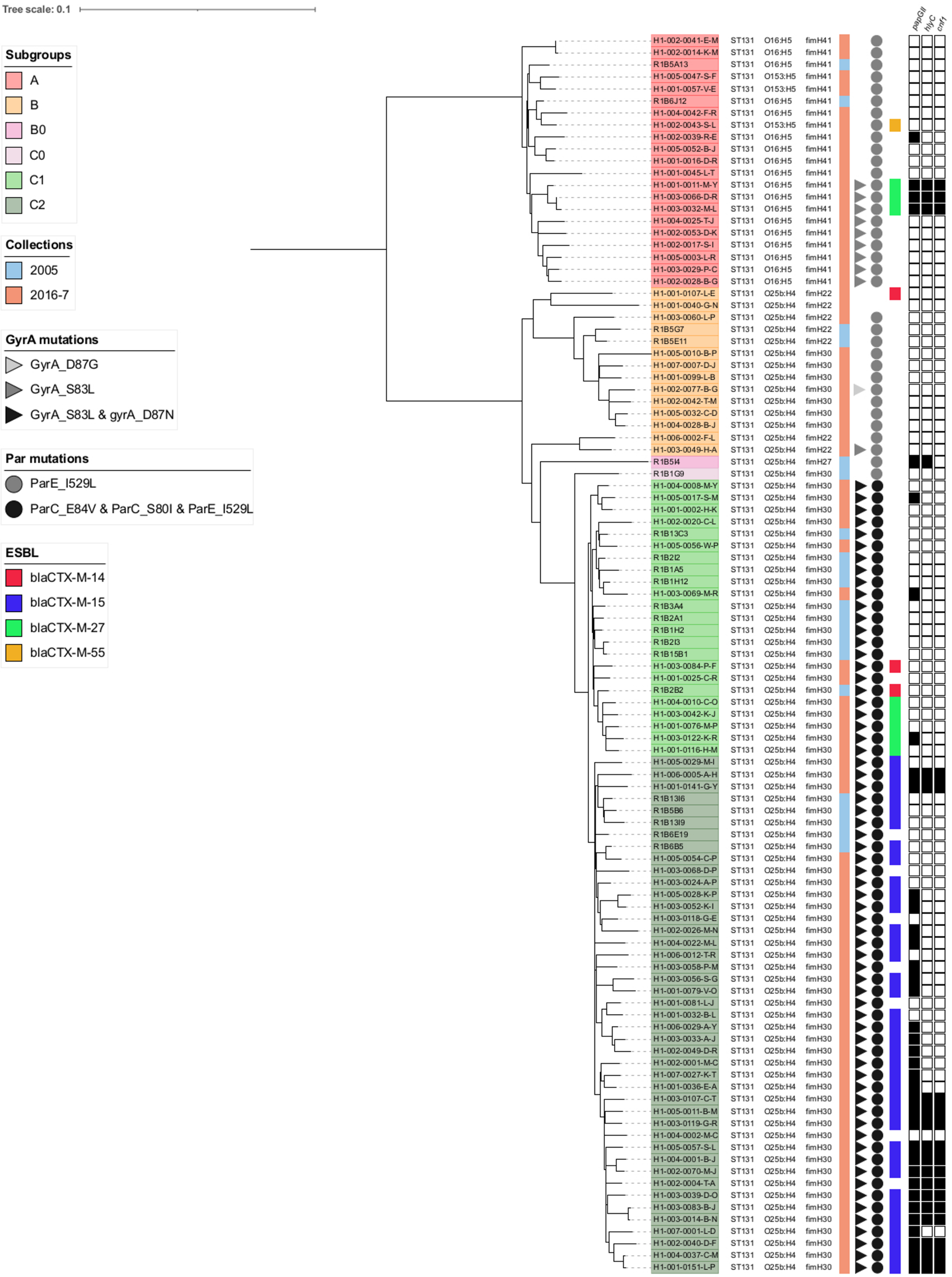

Figure S3. SNP-based phylogenetic tree of STc131 strains. The previously described clades/sub-clades A, B, B0, C0, C1 and C2 are highlighted in color. Mutations in GyrA and ParC/ParE are reported with triangle and circle, respectively. *bla*CTX-M coding genes are shown with colored squares and presence of *papGII*, *hlyC* and *cnf1* with black squares. The tree is mid-point rooted. The scale represents genetic distances in nucleotide substitution per site.

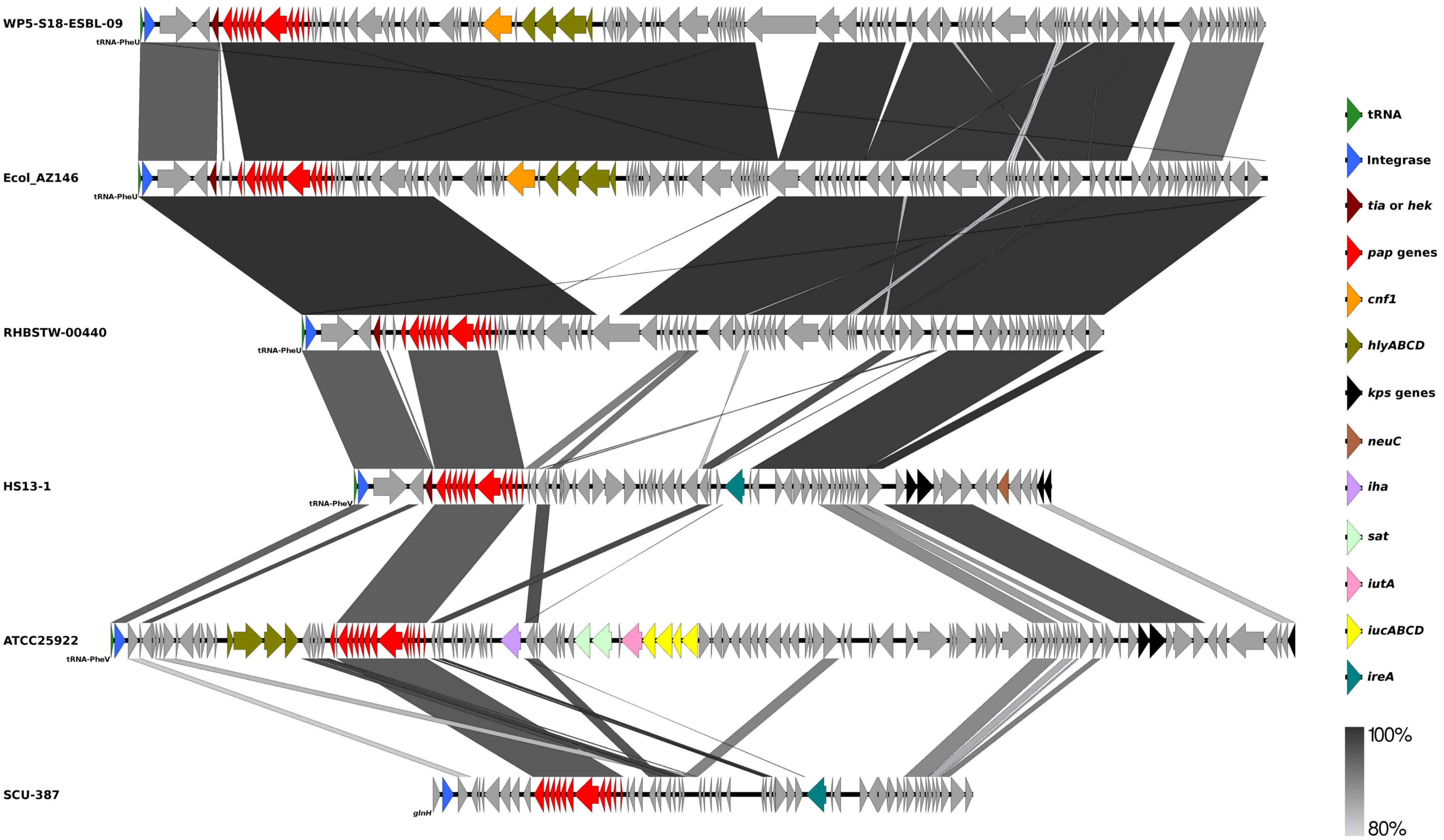

Figure S4. Genetic map of the reference PAIs found in the STc131 strains. The virulence genes of interest are highlighted in color as well as tRNA-PheU and tRNA-PheV. The result of the BlastN comparison between PAI is represented by grey to black blocks depending on the shared similarity of the sequences.

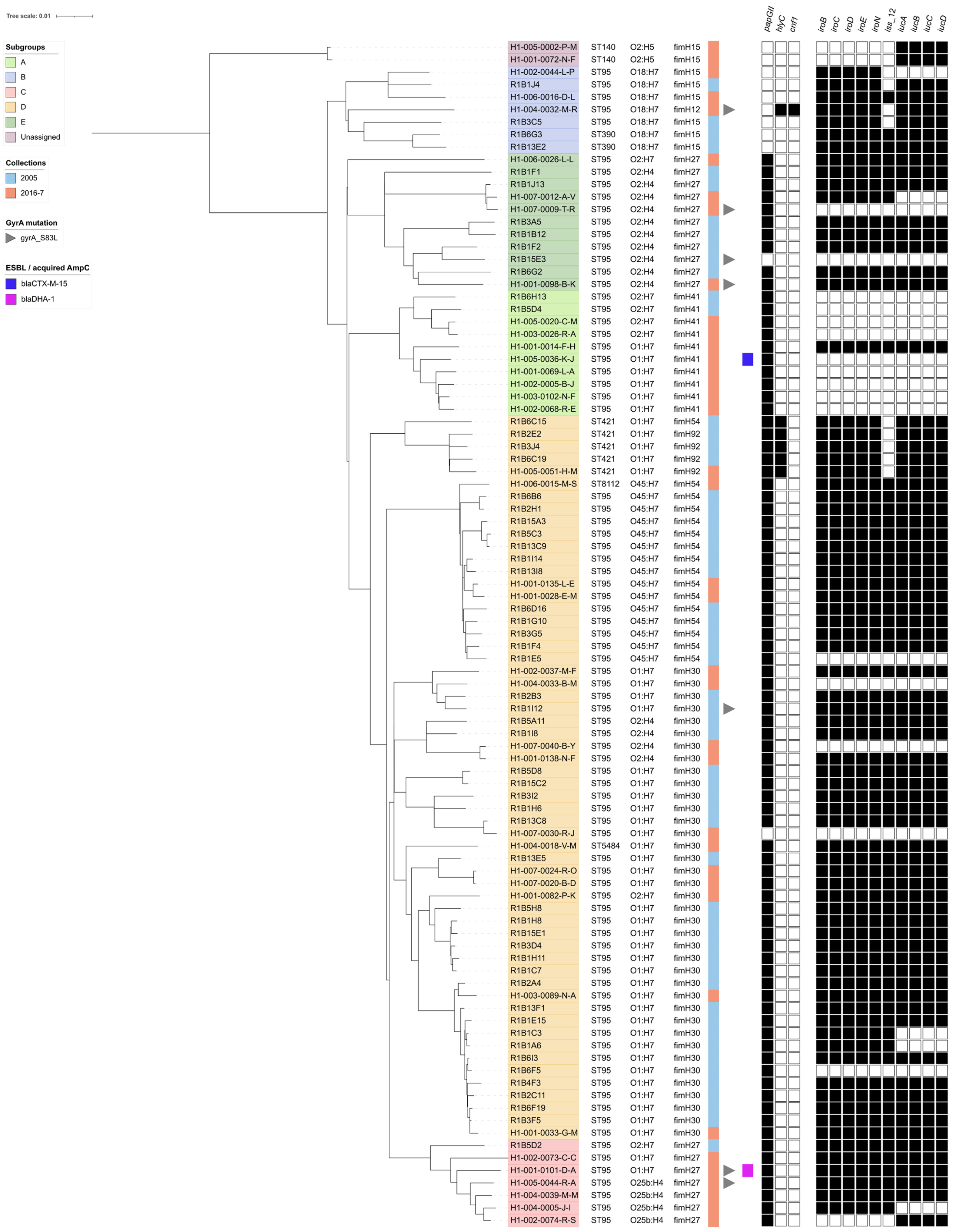

Figure S5. SNP-based phylogenetic tree of STc95 strains. The previously described subgroups A, B, C, D and E are highlighted in color. Mutations in GyrA are reported with triangle. ESBL and acquired AmpC coding genes are shown with colored squares and presence of *papGII*, *hlyC*, *cnf1*, *iro*B/C/D/E/N, *iss*_12 and *iuc*A/B/C/D with black squares. The tree is mid-point rooted. The scale represents genetic distances in nucleotide substitution per site.

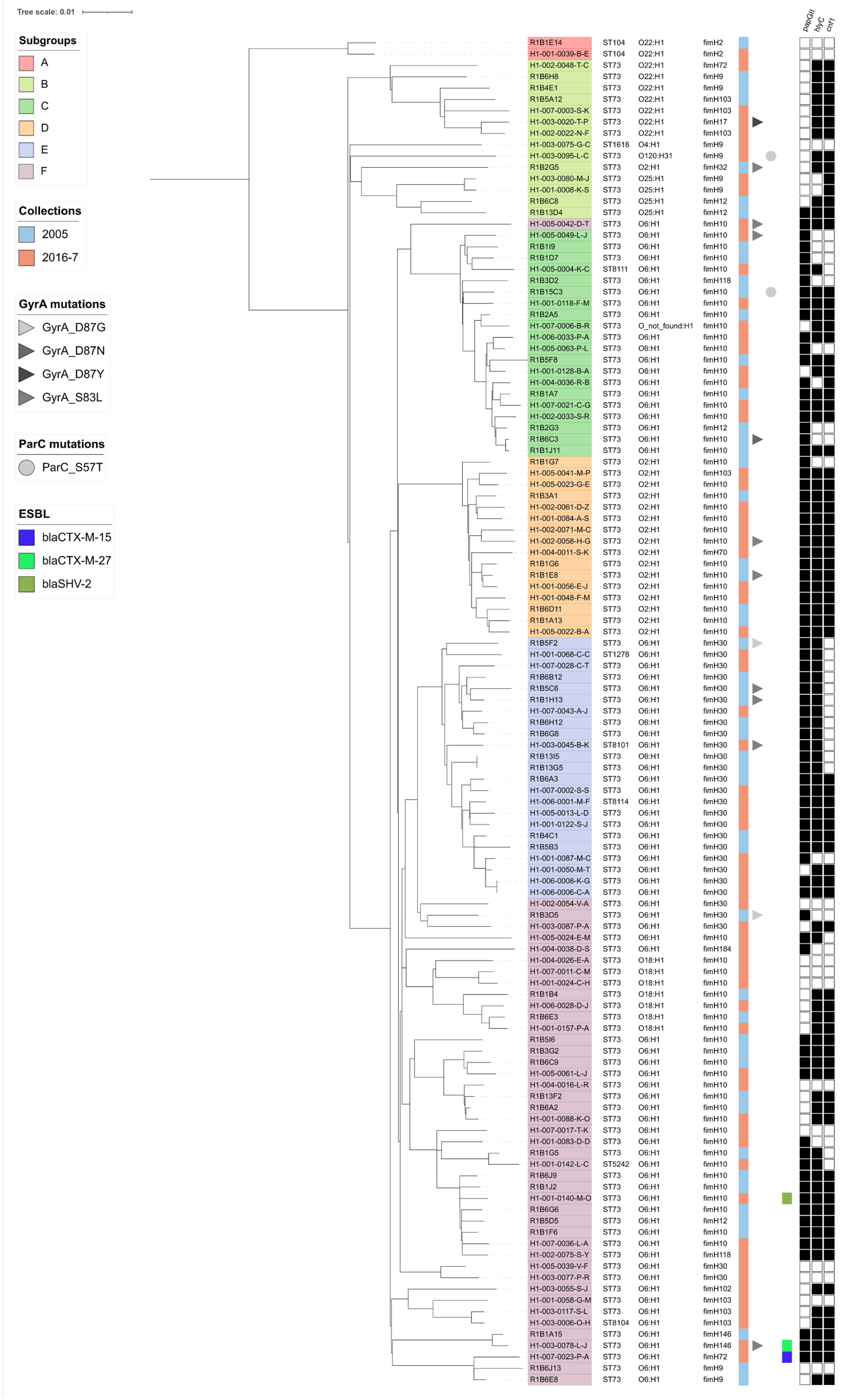

Figure S6. SNP-based phylogenetic tree of STc73 strains. The subgroups A to F, defined using fastbaps, are highlighted in color. Mutations in GyrA and ParC/ParE are reported with triangle and circle, respectively. ESBL coding genes are shown with colored squares and presence of *papGII*, *hlyC and cnf1* with black squares. The tree is mid-point rooted. The scale represents genetic distances in nucleotide substitution per site.

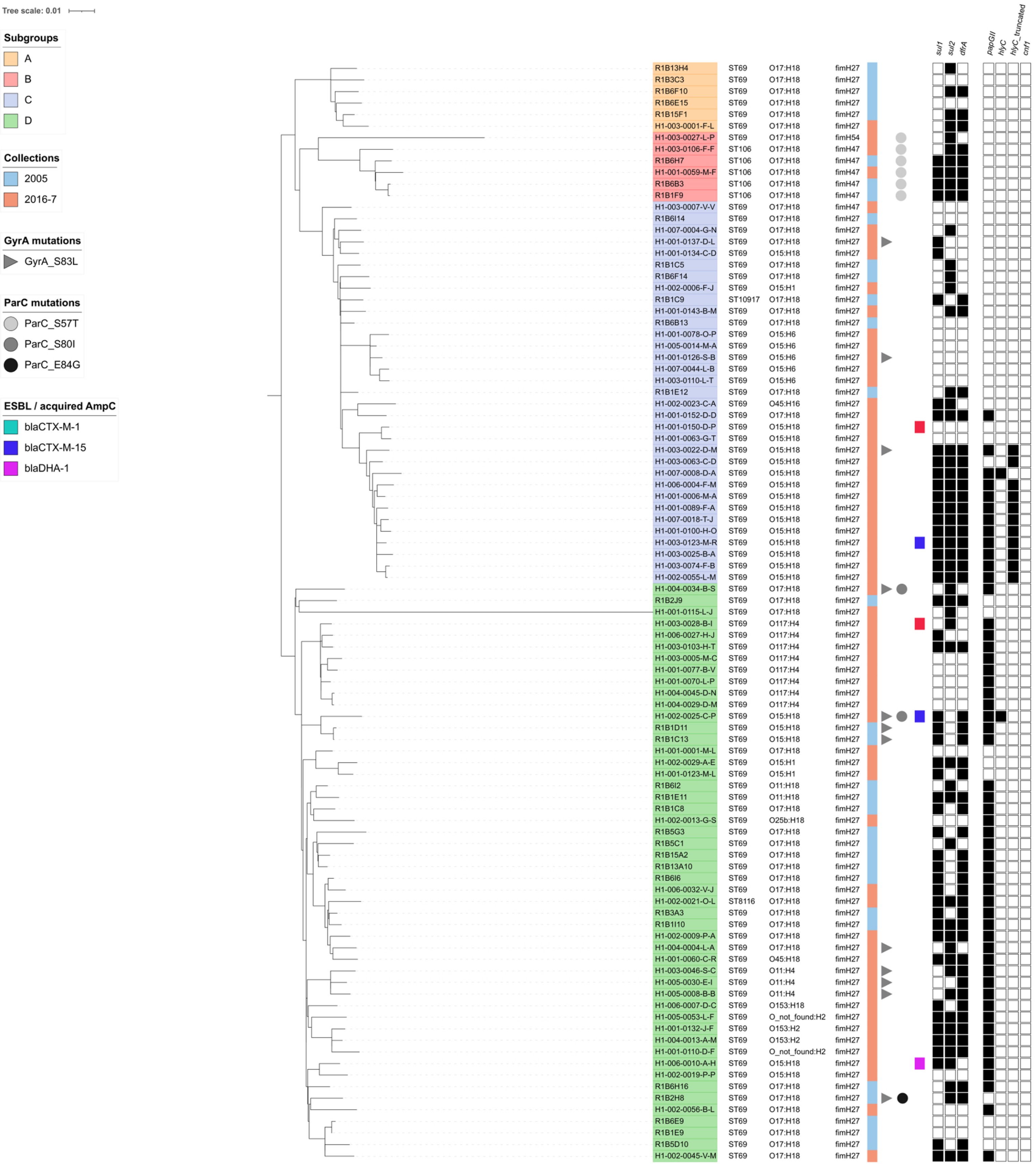

Figure S7. SNP based phylogenetic tree of STc69 strains. The subgroups A to D, defined using fastbaps, are highlighted in color. Mutations in GyrA and ParC/ParE are reported with triangle and circle, respectively. *bla*CTX-M coding genes are shown with colored squares. The presence of *sul1*, *sul2, dfrA, papGII, hlyC, hlyC* truncated and *cnf1* is shown with black squares. One strain (H1-001-0115-L-J) exhibits a very long branch, which could be related to a mutator phenotype as a non-synonymous mutation was found in MutS (A60T). The tree is mid-point rooted. The scale represents genetic distances in nucleotide substitution per site.

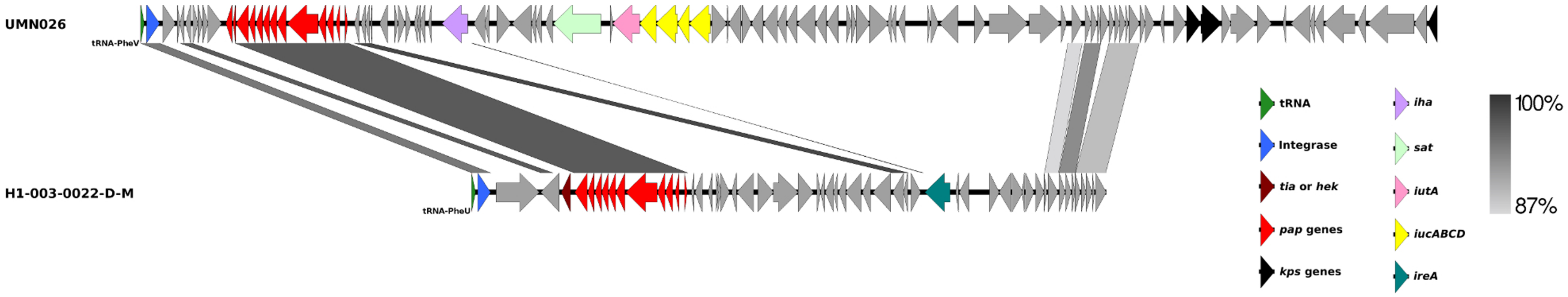

Figure S8. Genetic map of the reference PAIs found in the STc69 strains. The virulence genes of interest are highlighted in color as well as tRNA-PheU and tRNA-PheV. The result of the BlastN comparison between PAI is represented by grey to black blocks depending on the shared similarity of the sequences.

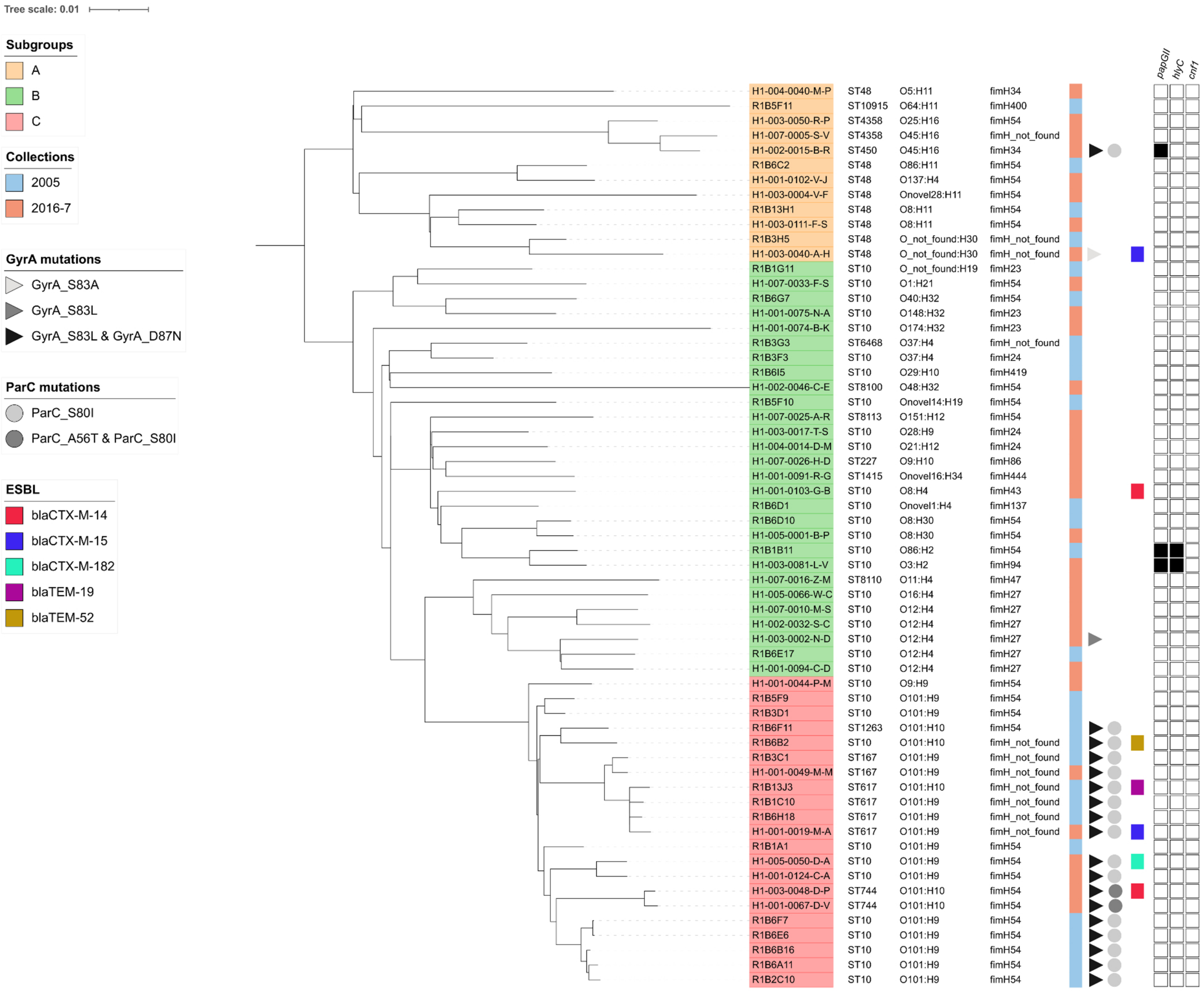

Figure S9. SNP-based phylogenetic tree of STc10 strains. The subgroups A to C, defined using fastbaps, are highlighted in color. Mutations in GyrA and ParC/ParE are reported with triangle and circle, respectively. ESBL coding genes are shown with colored squares and presence of *papGII*, *hlyC and cnf1* with black squares. The tree is mid-point rooted. The scale represents genetic distances in nucleotide substitution per site.
